# Dual targeting of the UPS and autophagy as a novel therapy for neurodegenerative proteinopathies

**DOI:** 10.3389/fncel.2026.1738415

**Published:** 2026-02-18

**Authors:** Georgie Lines, Martin Helley, Sébastien Gillotin, Janet Brownlees, James Duce, Phillip Smethurst

**Affiliations:** MSD (UK) Limited, London, United Kingdom

**Keywords:** autophagy, neurodegenearation, neurodegenerative diseases, proteasome, therapeutics, UPS—ubiquitin proteasome system

## Abstract

Neurodegenerative proteinopathies are characterized by impaired protein clearance and the accumulation of misfolded or aggregated proteins, ultimately leading to neuronal death. The two principal pathways responsible for protein degradation in cells are the ubiquitin proteasome system (UPS) and autophagy. Emerging evidence indicates that these pathways share regulatory components and engage in extensive crosstalk. In this review, we summarize the mechanisms of the UPS and autophagy, highlight their points of interaction, and discuss therapeutic opportunities to modulate both systems in parallel to enhance protein clearance in neurodegenerative disease.

## Proteostasis and neurodegenerative diseases

Proteostasis is the tightly controlled integration of multiple cellular processes that regulate the synthesis, folding and degradation of proteins (Hipp et al., 2019). A balanced proteome is essential for cell health, with degradation of proteins being critical for controlling cell signaling pathways and removing damaged or toxic proteins from the cellular environment (Damgaard, 2021). The two predominant mechanisms of protein degradation in mammalian cells are the ubiquitin proteasome system (UPS) and autophagy.

It is well established that the efficiency of both the UPS, and autophagy declines during normal aging and in numerous neurodegenerative diseases, including Alzheimer’s diseases (AD), Huntington’s disease (HD), Parkinson’s Disease (PD) and Amyotrophic lateral sclerosis and Frontotemporal dementia (ALS/FTD) (Keller et al., 2000; Munkácsy et al., 2019; Kelmer Sacramento et al., 2020; Saez and Vilchez, 2014; Seo et al., 2004; Mcnaught et al., 2003; Kabashi et al., 2012; Metaxakis et al., 2018; Nixon et al., 2005; Martin et al., 2015). These neurodegenerative diseases are characterized by an accumulation of pathological protein aggregates, which are not degraded by failing protein degradation systems. Accumulation of these toxic protein aggregates leads to disruption of cell homeostasis and toxicity in numerous brain cell types (Kumar et al., 2016; Tsoi et al., 2023).

AD is the most prevalent form of the neurodegenerative proteinopathies, accounting for up to 80% of all dementia cases. Individuals with AD demonstrate an accumulation of insoluble extracellular amyloid-beta plaques and intracellular tau tangles in the cortex and hippocampus which cause disruptions to cell health and eventually lead to cell death (Abeysinghe et al., 2020). HD is characterized by the aggregation of mutant huntingtin (mHTT), predominantly in the striatum (Jiang et al., 2023), while accumulation of alpha-synuclein in the Substantia nigra is indicative of Parkinson’s diseases (PD) (Tanner and Ostrem, 2024). ALS and FTD are characterized by accumulation of SOD1, FUS, tau, and TDP-43, and due to significant overlap of pathological molecular mechanisms, these diseases are considered to be part of the same spectrum (Verde et al., 2025). Causative mutations have been identified in familial cases of these neurodegenerative diseases, some of which are associated with protein degradation systems. For example, mutations of VCP and p62 in ALS/FTD, PINK1/PARKIN mutations in PD and variation in the TRIM11 locus in progressive supranuclear palsy (PSP) (Johnson et al., 2010; Jabbari et al., 2018; Wasner et al., 2020). There is also growing evidence of co-pathology with respect to aggregated proteins where aggregation of proteins such as tau are implicated in multiple different neurodegenerative diseases (Samudra et al., 2023), indicating that reduced proteostasis is a common pathogenic mechanism across these disorders (Di Domenico and Lanzillotta, 2022).

As a result of the increasing evidence linking decreased proteostasis and neurodegeneration, boosting the activities of protein degradation systems, like the UPS and autophagy, as a treatment for these diseases has emerged as a promising therapeutic strategy (Rao et al., 2015; Cui et al., 2022). Many studies have focused solely on one degradation mechanism, however in the last decade it has become evident that the UPS and autophagy are heavily interconnected, with multiple reports of cross talk and co-regulation between the two (Yang et al., 2013; Ji and Kwon, 2017; Kocaturk and Gozuacik, 2018). Indeed, upregulation of autophagy would be beneficial to increase the degradation of insoluble protein aggregates, which are too large to be degraded by the UPS. At the same time, the simultaneous upregulation of UPS activity could be beneficial in increasing the degradation of smaller intrinsically disordered proteins that are susceptible to misfolding and aggregation and further reducing or preventing further seeded aggregation and cell-to-cell spread of pathology. Therefore, targeting shared pathways of these two systems to concurrently promote their degradation activity may be a more effective way of increasing the overall capacity of cells to degrade pathological proteins.

Here, we review the mechanisms of the UPS and autophagy, the cross talk between the two systems, and highlight pathways involved in both mechanisms that could be modulated to increase cellular proteostasis as a novel treatment for neurodegenerative diseases.

## The ubiquitin proteasome system

**FIGURE 1:**
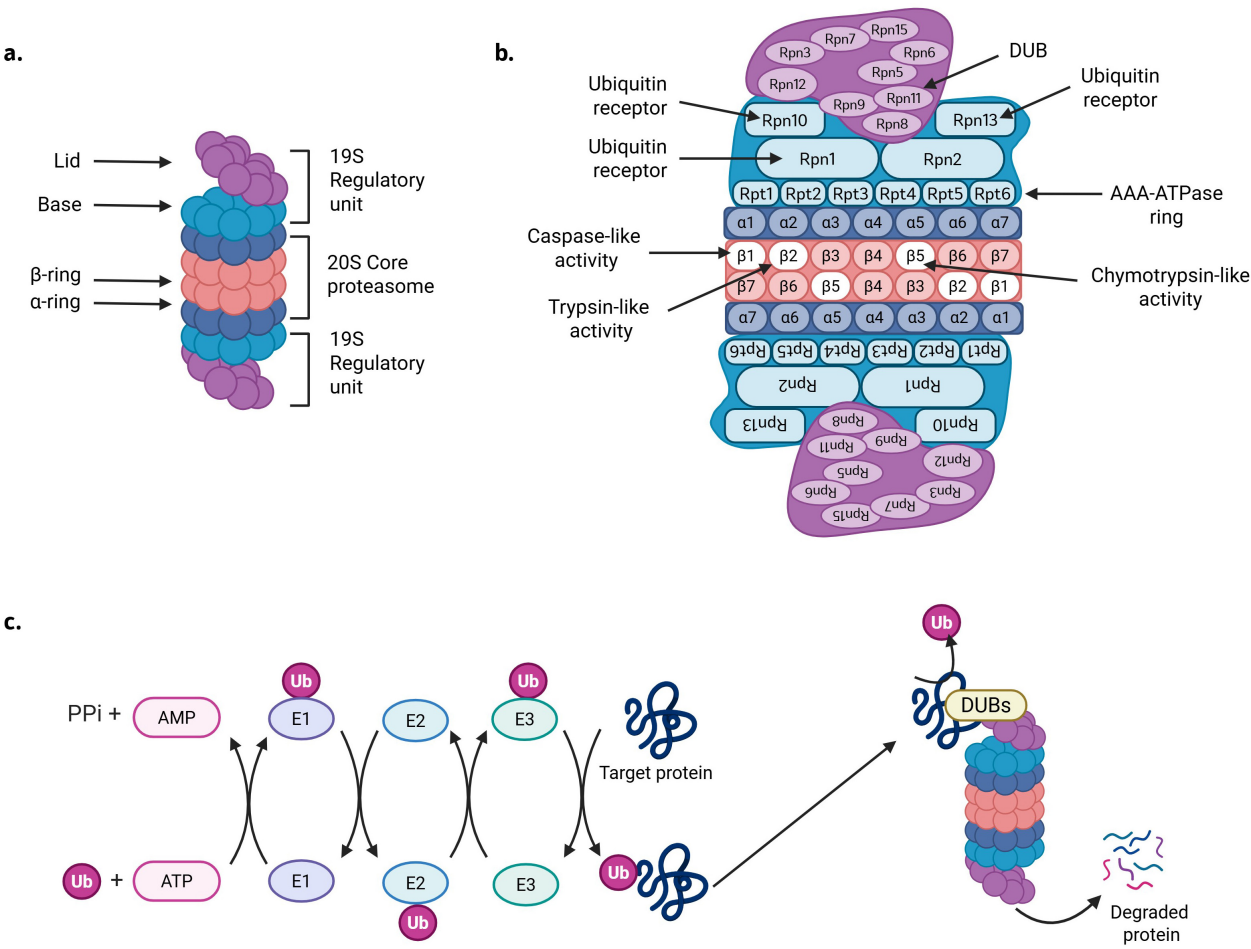
Protein degradation by the ubiquitin proteasome system. **(a)** Overview of the structure of the 30S proteasome, formed of a 20S core unit capped both ends with a 19S regulatory unit. The 20S unit is formed of two α and two β rings. The 19S subunit is split unto a lid and a base unit. **(b)** Detailed view of the subunits that comprise the 30S proteasome, and their functional roles. **(c)** Overview of the ubiquitination of a substrate protein targeted for degradation by the proteasome. Briefly, ubiquitin is activated by ubiquitin-activating enzyme E1. Activated ubiquitin is transferred to E2, a ubiquitin conjugating enzyme. In the final step, ubiquitin is conjugated to the target protein via an E3 ubiquitin protein ligase enzyme. After ubiquitination and delivery to the proteasome, the target protein is deubiquitinated by DUBs and degraded. Created in BioRender.com.

The proteasome is a dynamic and highly conserved multi-unit protein complex that has been reported to be responsible for around 80–90% of cellular protein turnover via the UPS (Lee and Goldberg, 1998; Yu and Hua, 2023). A variety of proteasome conformations exist within mammalian cells which are variations and extensions of the constitutive 20S core proteasome (CP). The 20S CP, which comprises around 1% of total cellular proteins, is formed of 4 stacked heptameric rings (two α-rings and two β-rings) that enclose and gate a central proteolytic chamber (Figure 1a). The two alpha rings are formed from subunits α1-α7, and the two inner β-rings are formed of subunits β1-β7. In the constitutively expressed 20S CP, each of the two inner β-rings contains 3 proteolytic subunits: β1, β2, and β5 which possess caspase-like, trypsin-like, and chymotrypsin-like activity, respectively (Figure 1b).

The majority of proteins targeted to the proteasome for degradation are cleared in a ubiquitin dependent manner via 26S or 30S proteasomes, also referred to as singly, or doubly capped proteasomes, respectively, which are localized to the cytoplasm (Tanaka, 2009). As the name suggests, these proteasome variations consist of a central 20S CP, capped on one (26S) or both (30S) ends with a 19S regulatory unit (Figure 1a). The 19S unit is formed from 19 subunits, which are categorized into a lid and a base (Glickman et al., 1998). The base of the 19S subunit consists of four regulatory particle non-ATPase (Rpn) subunits: Rpn1, Rpn2, Rpn10 and Rpn13, as well as six regulatory particle AAA-ATPase (Rpt) subunits, Rpt1–6, collectively referred to as the AAA-ATPase ring. The 19S lid complex is formed from Rpn3, Rpn5–9, Rpn11, and Rpn12 (Sharon et al., 2006; Figure 1b).

In the cytoplasm, proteins are targeted for degradation by the 26S/30S proteasome via the attachment of polyubiquitinated chains. E1, E2, and E3 enzymes work in consort to conjugate ubiquitin to the target protein. Briefly, a specific ubiquitin-activating enzyme, E1, activates the c-terminal glycine residue of ubiquitin in an ATP-dependent reaction (Haas et al., 1982; Pohl and Dikic, 2019). Following activation, ubiquitin is transferred to a cysteine residue of E2, a ubiquitin conjugating enzyme (Tai and Schuman, 2008). In the third step, E3, a ubiquitin-protein ligase mediates the conjugation of ubiquitin to a lysine residue of the target protein (Pohl and Dikic, 2019; Figure 1c). In humans up to 8 different E1, 40 E2 and 600 E3 enzymes have been reported (Groettrup et al., 2008; Stewart et al., 2016; Tieroyaare Dongdem and Adiyaga Wezena, 2022). The larger number of E3 ligases allows for determining the specificity of protein ubiquitination. Substrates targeted toward degradation via the proteasome are predominantly tagged with K48-linked ubiquitin chains, however, K11 and K63 ubiquitin linkages have also been reported to facilitate proteasomal protein degradation (Dwane et al., 2017; Ji and Kwon, 2017).

The 19S lid unit of the proteasome recognizes polyubiquitinated proteins via its ubiquitin receptor subunits, Rpn1, Rpn10 and Rpn13 (Deveraux et al., 1994; Husnjak et al., 2008; Schreiner et al., 2008; Shi Y. et al., 2016). Rpn10 predominantly binds K48-linked chains through its ubiquitin interacting motif (UIM), whereas Rpn1 binds ubiquitin via its torid repeat region (Deveraux et al., 1994; Shi Y. et al., 2016; Martinez-Fonts et al., 2020). It’s been reported that Rpn10 and Rpn1 can work together as co-receptors to recognize K63-linked chains and monoubiquitinated substrates (Martinez-Fonts et al., 2020). Rpn13 binds ubiquitin via its pleckstrin-like ubiquitin domains (Husnjak et al., 2008; Schreiner et al., 2008). After substrate recognition, the protein is deubiquitinated via the proteasomes intrinsic deubiquitinating enzyme (DUB), Rpn11 (Maytal-Kivity et al., 2002; Verma et al., 2002; Bard et al., 2018). Other reversibly associated DUBs can aid in selecting proteins for degradation, for example ubiquitin specific peptidase 14 (USP14) and ubiquitin C-terminal hydrolase 37 (UCH37, also known as UCHL5) (Lee M. J. et al., 2011). Once recognized and deubiquitinated, the target protein is unfolded and pulled into the central 20S CP chamber via the AAA-ATPase ring in a process that requires ATP (Nyquist and Martin, 2014). The proteasome cleaves substrates into short chains of 3–25 amino acids, which are subsequently cleaved to single amino acid fragments by cytoplasmic peptidases. This allows for cellular recycling of amino acids to build new proteins (Kocaturk and Gozuacik, 2018).

The uncapped 20S proteasome can exist in isolation and has been reported to be localized to both the nucleus and the cytoplasm (Baumeister et al., 1998). Unlike the 26S/30S proteasome, the 20S can directly degrade misfolded, oxidatively damaged and intrinsically disordered or unstructured proteins in a ubiquitin-independent manner (Raynes et al., 2016). The 20S core exists mainly in a closed state whereby the N-terminal tails of the alpha subunits converge to form a gate to the proteolytic chamber. When a target protein binds to the 19S regulatory caps of 26S/30S proteasome conformation, this induces a conformational change that results in the 20S CP opening and protein degradation. On the contrary, gate opening in uncapped 20S proteosomes is thought to be induced by direct binding of the target protein to the CP (Ukmar-Godec et al., 2020). The 20S CP can also associate with non-ATP regulatory protein complexes such as the 11S complex (PA28) and Blm10 (PA200) which can induce 20S gate opening and protein degradation in an ATP-independent manner (Whitby et al., 2000; Sadre-Bazzaz et al., 2010). Reports have suggested that the 20S CP may be responsible for the degradation of up to 20% of cellular proteins (Baugh et al., 2009).

Interestingly, recent work by Ramachandran and colleagues reported a mammalian nervous system specific plasma membrane imbedded proteasome complex, that has access to both inter and extracellular compartments. The neuronal membrane proteasome (NMP) is consistent with the 20S CP and contains the standard β1, β2, β5 subunits. Intracellular proteasome inhibition blocked the release of extracellular peptides and reduced neuronal calcium signaling, suggesting the NMP is important for cleaving and releasing peptides required for cell signaling (Ramachandran and Margolis, 2017).

During oxidative stress and under pro-inflammatory stimuli, 3 alternative proteasome subunits LMP2(β1i), MECL-1 (β2i), and LMP7 (β5i) are preferentially incorporated into 20S proteasomes forming the immunoproteasome (i20S) (Aki et al., 1994; Meiners et al., 2014). Incorporation of these alternative subunits alters the substrate binding pocket of the proteasome, making the i20S more effective at antigen processing for presentation on MHC class molecules and signaling inflammatory responses (Murata et al., 2018; Basler et al., 2013; Huber et al., 2012; Sijts et al., 2000; Toes et al., 2001). Further, the i20S can associate with the IFN-y-inducible PA28 on one or both ends of its barrel, which may further aid in ubiquitin independent protein degradation (Rechsteiner et al., 2000).

A further variation of the β5 catalytic subunit, β5t, was identified in cortical thymic epithelial cells (cTECs). β5t, substitutes β5 or β5i in the standard or immunoproteasome to form the Thymoproteasome. Current understanding of this substitution is still limited, but studies have demonstrated that the β5t subunit has a reduced chymotrypsin like activity compared to β5 and β5ti (Murata et al., 2018; Tanaka, 2009).

## Autophagy

**FIGURE 2:**
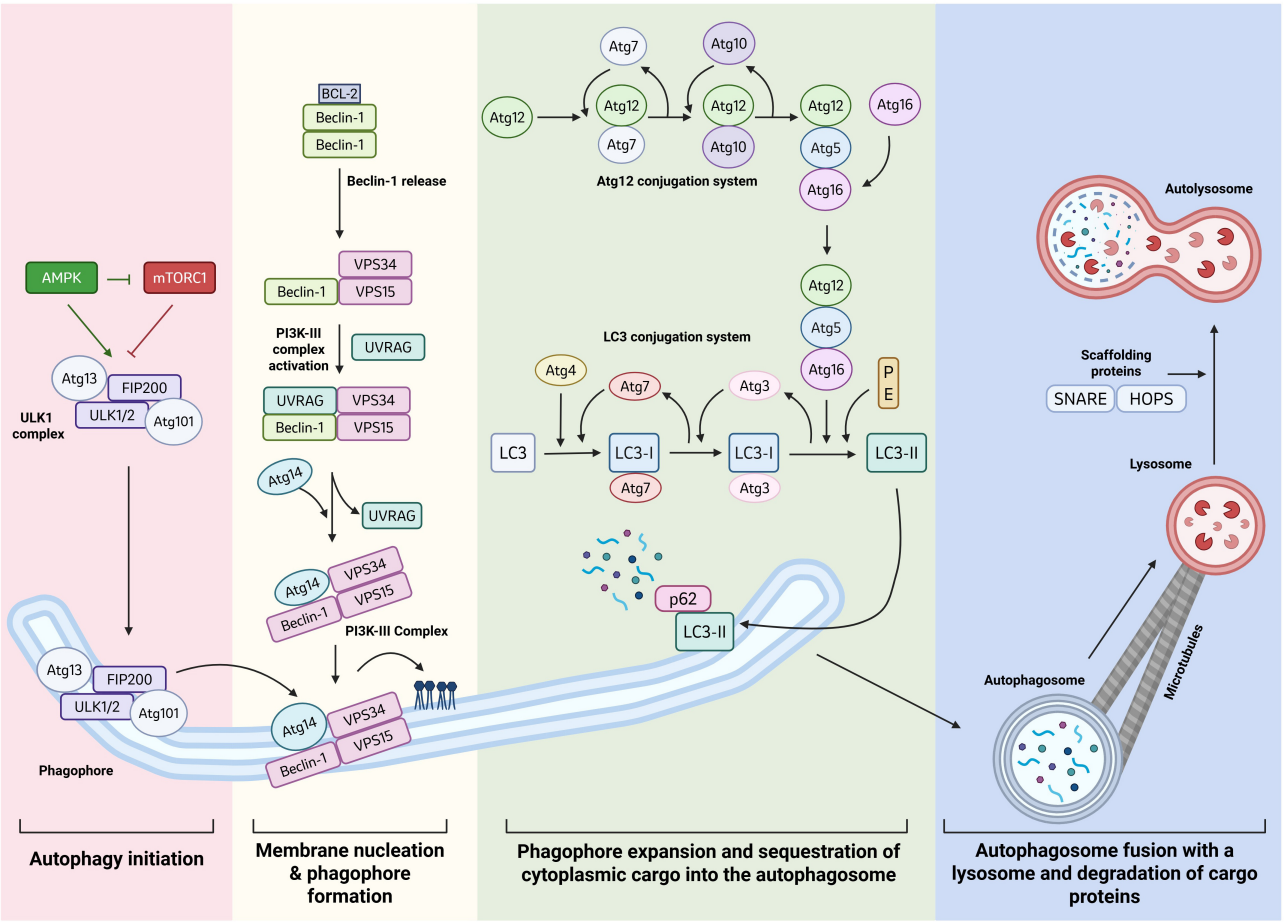
Protein degradation via autophagy. AMPK activation, or mTOR inhibition leads to the activation of the ULK1 complex. Activated ULK1 complex recruits the PI3K-III complex which aids in the elongation of the phagophore membrane. The PI3K complex is regulated by Beclin-1 release from BCL-2 and activation via UVRAG. LC3 is processed to LC3-II through an E1/E2/E3-like system involving multiple Atg proteins. LC3-II is localized to both the inner and outer membrane of the growing phagophore where it acts as an autophagy cargo receptor, trapping proteins within the autophagosome for degradation. After closing of the phagophore membrane and maturation into an autophagosome, the autophagosome fuses with a lysosome to form an autolysosome. Proteins within the autolysosome are degraded by proteases and cathepsins. Created in BioRender.com.

Three primary types of autophagy have been described in mammalian cells, macroautophagy, chaperone mediated autophagy (CMA) and microautophagy (Yamamoto and Matsui, 2024; Vargas et al., 2023). In this review, we will briefly discuss the best characterized type of autophagy, macroautophagy (herein referred to as autophagy). The process of autophagy can be broken down into 4 basic steps: (1) initiation, (2) membrane nucleation and phagophore formation, (3) phagophore expansion and sequestration of cargo and (4) fusion with a lysosome and protein degradation (Figure 2). Autophagy has been reported to be responsible for around 10–20% of total protein degradation in the cell (Lilienbaum, 2013; Clague and Urbé, 2010; Yu and Hua, 2023).

mTOR complex 1 (mTORC1) and AMP activated protein kinase (AMPK) are key regulators of autophagy initiation (Kamada et al., 2000). mTORC1 is formed of three core proteins, mTOR (mammalian target of rapamycin), mLST8 (mammalian lethal with SEC13 protein 8) and Raptor (regulatory-associated protein of mTOR) (Hennig et al., 2021). In the presence of growth factors and nutrients, mTORC1 inhibits autophagy initiation by phosphorylating the ULK1 complex, formed of Unc-51 protein kinase (ULK1), FAK—family interacting protein 200 (FIP200), and autophagy related gene proteins, Atg13 and Atg101 (Metaxakis et al., 2018; Hara et al., 2008; Chan et al., 2009; Ganley et al., 2009; Hosokawa et al., 2009b,Jung et al., 2009; Kim et al., 2011; Figure 2). This inhibitory phosphorylation by mTOR occurs at S638 and S758 of ULK1 and S258 of Atg13 (Puente et al., 2016; Shang et al., 2011; Mercer et al., 2018). Upon nutrient depletion, autophagy is activated to degrade unnecessary or defunct proteins in order to release and recycle nutrients to meet the cells metabolic needs (Russell et al., 2014). A reduction in cellular ATP triggers the activation of AMPK which initiates membrane nucleation by inhibiting mTORC1 (Ke et al., 2018; Mercer et al., 2018). mTOR-dependent phosphorylation sites are dephosphorylated, and the ULK1 complex autophosphorylates itself as well as phosphorylating FIP200 and Atg13 (Alers et al., 2012; Ganley et al., 2009; Hosokawa et al., 2009a,Zachari and Ian, 2017). Further, AMPK activates the ULK1 complex through direct phosphorylation at S317 and S777 of ULK1 (Kim et al., 2011).

Autophagy regulation and initiation is complex, and in addition to phosphorylation, ubiquitylation has been reported to play a critical role in regulating autophagy initiation via ULK1 activation. AMBRA1 (Activating Molecule in Beclin1-regulated autophagy) interaction with E3 ligase TRAF6 (TNF receptor associated factor 6) has been reported to lead to K63-linked ubiquitination of ULK1, promoting its dimerization, stabilization, and activation (Nazio et al., 2013; Mercer et al., 2018). Jiao and colleagues reported that chaperone-like protein p32 also regulates ULK1 stability by binding ULK1 and promoting K63-linked ubiquitination over K48-linked ubiquitination, increasing ULK1 stability (Jiao et al., 2015). In contrast, under starvation conditions, the E3 ligases NEDD4L and Cullin-3 have been reported to restrict autophagy by promoting ULK1 degradation through K27 or K48-linked ubiquitination respectively (Nazio et al., 2016; Liu C. C. et al., 2016, Mercer et al., 2018).

Activated ULK1 complex accumulates in close proximity to the endoplasmic reticulum (Zachari and Ian, 2017) and recruits the PI3K complex, composed of vacuolar protein sorting homologs VPS34 and VPS15, Atg14L and Beclin 1 (Russell et al., 2014; Itakura et al., 2008; Matsunaga et al., 2010; Figure 2). VPS34, a phosphoinositide-3-kinase, generates phosphatidylinositol-3-phosphate (PI3P) from phosphatidylinositol (PIP) (Liu et al., 2023). PI3P is essential for elongating and expanding the growing phagophore membrane, alongside Atg9 which aids in recruiting the required lipids (Holzer et al., 2024). To form the PI3K complex, Beclin-1 must be released from BCL-2, which can hold Beclin-1 in an inhibited dimer state. Following Beclin-1 release and formation of the early PI3K complex (VPS34, VPS15 and Beclin-1), UVRAG (UV radiation resistance associate gene) binds and activates the complex, before disassociating again to allow ATG14 addition (Figure 2). Elongation of the growing autophagosome membrane is dependent on two ubiquitin-like conjugation systems. Firstly, Atg12 is conjugated to Atg5 via Atg7 which acts in a E1-like capacity, and Atg10 which acts in a E2-like manner. Atg16L is recruited to the Atg12-Atg5 dimer to create a larger protein complex (Atg12-5-16L) which functions as an E3 ligase to conjugate lipids to Atg8/MT-associated protein 1 (LC3). Directly after its synthesis, LC3 is cleaved via Atg4 into LC3-I (Maruyama and Noda, 2018). LC3-I is conjugated with PE (phosphatidylethanolamine) into LC3-II, one of the most common biochemical markers of autophagy, via the Atg7 (E1-like), Atg3 (E2-like) and Atg12-5-16L complex (E3-like) cascade (Nishimura and Tooze, 2020; Kocaturk and Gozuacik, 2018; Figure 2). LC3-II is bound to both the inner and outer membrane of the growing phagophore where it functions as an adapter protein than can recruit selective cargo for degradation by interaction with cargo receptors such as p62, Nuclear Domain 10 protein 52 (NDP52), optineurin (OPTN) and Neighbor of BRCA gene1 (NBR1). These autophagy receptors possess ubiquitin binding domains (UBDs) and LC3 interacting regions (LIRs) to sequester substrates into growing autophagosomes (Dósa and Csizmadia, 2022; Cohen-Kaplan et al., 2016a,Johansen and Lamark, 2011; Figure 2). Autophagy substrates are typically decorated with K63-linked chains, however p62 has been reported to chaperone both K48 and K63-linked substrates to the autophagosome for degradation (Liu W. J. et al., 2016). In addition, K6 and K11 ubiquitination has also been associated with autophagic degradation (Dwane et al., 2017; Ji and Kwon, 2017; Riley et al., 2010).

Once the phagophore membrane closes, the vesicle matures into an autophagosome and the Atg5-Atg12-Atg16 complex dissociates. The autophagosome is trafficked to the site of lysosomes via microtubule retrograde transport, and fusion with a lysosome is assisted by attachment protein receptors and scaffolding complexes including SNAREs (Soluble NSF attachment protein receptors) and HOPS (homotypic fusion protein sorting complex) (Lőrincz and Juhász, 2020). The fusion of the autophagosome and lysosome creates an autolysosome (Figure 2). Protein cargo is degraded via proteases and cathepsins within the acidic environment of the autolysosome, which is maintained by V-type ATPase pumps that push protons into its lumen (Lőrincz and Juhász, 2020).

## Cross talk and co-regulation of the UPS and autophagy

Early observations established the conventional wisdom that the UPS and autophagy were two distinctly separate, but parallel mechanisms of protein degradation, with no common regulators or crosstalk between them (Korolchuk et al., 2010; Ciechanover et al., 1984; Pickart, 2004). This paradigm came to precedence due to the differing machinery and substrate preferences of the two degradation mechanisms. The UPS favors soluble short lived cytosolic proteins whose temporally regulated degradation is often essential for cell signaling pathways. In contrast, degradation via autophagy occurs within autolysosomes, not the cytosol, and was classically reported as a method of non-selective bulk degradation of larger aggregated proteins and damaged organelles, and a response to nutrient starvation (Kocaturk and Gozuacik, 2018). However, more recently, it has become evident that there is a significant amount of cross talk and co-regulation between the UPS and autophagy pathways, and the current understanding is that these two systems should be considered linked in a network that works to maintain cellular proteostasis through controlled protein degradation (Ji and Kwon, 2017; Kocaturk and Gozuacik, 2018; Lilienbaum, 2013; Cohen-Kaplan et al., 2016a,Raffeiner et al., 2023).

## Regulation of autophagy by the UPS

The UPS is reported to directly regulate autophagy flux through controlled degradation of several key autophagy machinery components. Firstly, LC3 is degraded in a stepwise manner by the 20S proteasome, and this can be reduced by p62 binding to the LC3 N-terminus (Gao et al., 2010). In addition, Beclin-1 can be labeled for proteasomal degradation either through K48-linked ubiquitylation by E3 ligase RNF216 (Xu et al., 2014), or K11 ubiquitylation via E3 ligase NEDD4 which blocks its ability to interact with VPS34, promoting destabilization and degradation via the proteasome (Platta et al., 2012). Indeed, the ubiquitination and subsequent proteasomal degradation of the autophagy regulator AMBRA1 is also controlled by its dynamic interaction with the E3 ligase Cullin-4, which in turn limits AMBRA1 abundance and modulates autophagy (Antonioli et al., 2014). Lastly, Njomen and colleagues have shown that chemical activation of the 20S proteasome can promote the degradation of proteins involved in autophagosome-lysosome fusion, such as SNAP29 and STX17, resulting in reduced autolysosome biogenesis (Njomen and Tepe, 2019).

In multiple cellular models, disruption to proteasome activity (e.g., with small molecule inhibition or genetic modulation) leads to a compensatory upregulation of autophagy activity (Zhu et al., 2010; Wu et al., 2008; Selimovic et al., 2013; Demishtein et al., 2017; Ge et al., 2009; Kyrychenko et al., 2014). A number of pathways have been proposed to contribute toward crosstalk from the UPS to autophagy including the unfolded protein response (UPR) (Hetz et al., 2015), N-terminal arginylation of the N-end rule pathway (Cha-Molstad et al., 2017) and p53 (White, 2016).

**FIGURE 3:**
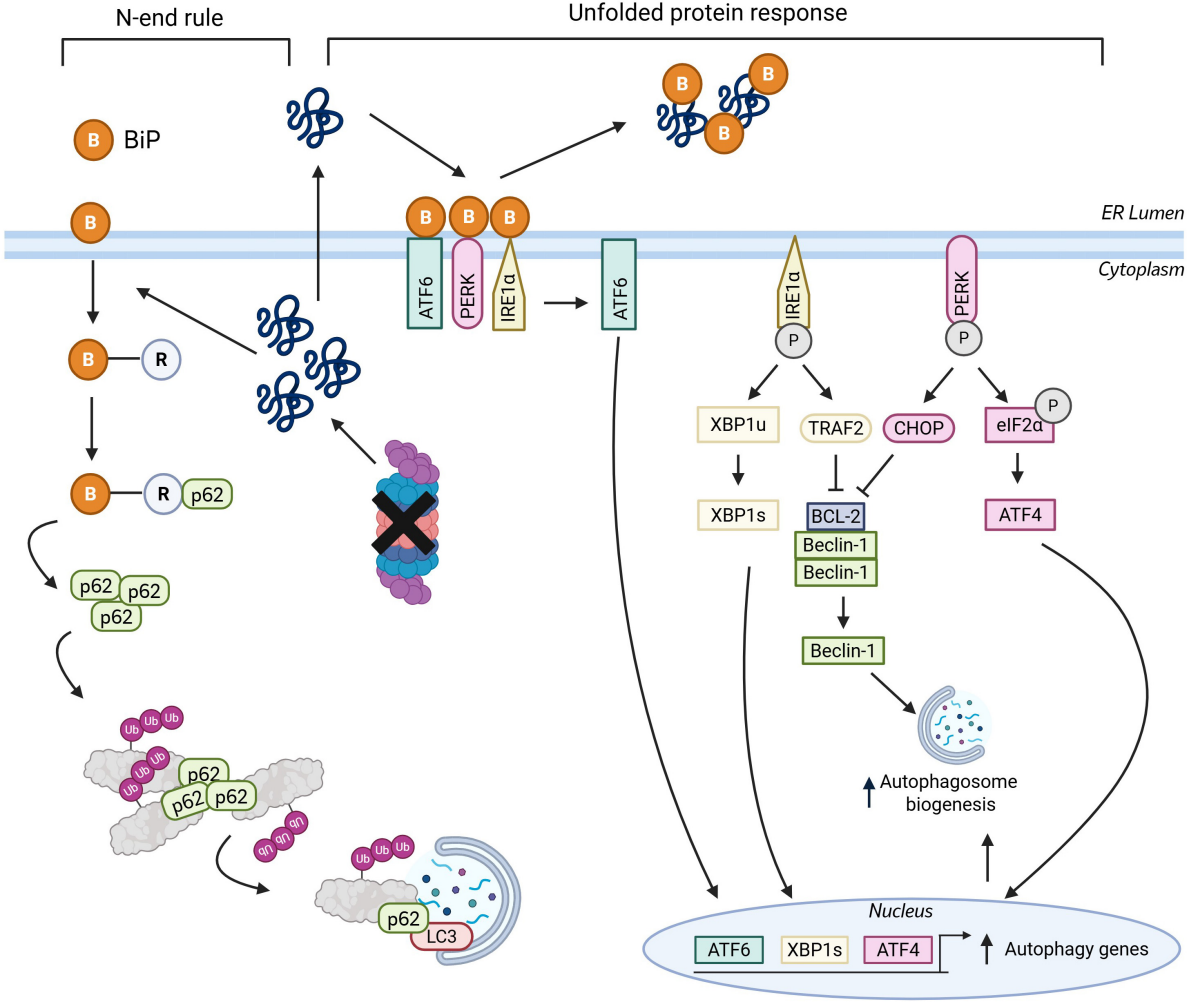
The N-end rule and unfolded protein response and the in crosstalk from the UPS to autophagy. Proteasomal inhibition leads to a build-up of misfolded or aggregated proteins in the cytoplasm and the ER. In the N-end pathway, BiP is relocated to the cytoplasm, where it is arginylated. Arginylated BiP binds to p62, leading to its oligomerization and increased efficiency at sequestering proteins to target them toward degradation via autophagy. An accumulation of misfolded proteins in the ER lumen triggers the unfolded protein response, whereby BiP disassociates from membrane receptors PERK, IRE1α and ATF6, leading to receptor activation. Activation of the PERK-eiF2α pathway leads to the increased expression of transcription factor ATF4, which in turn leads to the upregulation of autophagy associated genes. In the IRE1α-TRAF2 pathway, splicing of XBP1u (unspliced) into XBP1s (spliced) leads to increased autophagy gene expression. TRAF2 inhibits BCL-2, which leads to its release of Beclin-1 and increase autophagosome biogenesis. Activation of the ATF6 pathway leads to increase autophagy gene expression through ATF6 translocation to the nucleus and transcriptional activator activity. B = BiP, R = Arginylation. Created in BioRender.com.

The UPR pathway appears to have a key role in modulating crosstalk between the UPS and autophagy. Proteasome inhibition leads to an accumulation of misfolded proteins in the lumen of the endoplasmic reticulum (ER), which in turn causes dissociation of chaperone binding immunoglobulin protein (BiP) from ER membrane receptors inositol requiring 1α (IRE1α), activating transcription factor 6 (ATF6) and PKR-like ER kinase (PERK) (Figure 3). Release of BiP activates these receptors to initiate downstream signaling toward autophagy upregulation, and in turn free BiP can bind to unfolded proteins to aid in their correct folding (Kocaturk and Gozuacik, 2018). The IRE1-TRAF2 pathway leads to the splicing of XBP1u to XBP1s which, upon translocation to the nucleus, increases the expression of autophagy associated genes. Further, activation of the IRE1 pathway leads to JNK phosphorylation. BCL-2, which is bound to Beclin-1 in non-starvation conditions to inhibit autophagy, is phosphorylated by p-JNK at residues T69, S70, and S87, to release it from Beclin-1 and thereby increasing autophagosome biogenesis (Deegan et al., 2013; Ji and Kwon, 2017; Wei et al., 2008). ATF6 also modulates autophagy by inducing the expression of DAPK1, which in turn phosphorylates Beclin-1 to increase autophagosome formation (Gade et al., 2012; Zalckvar et al., 2009; Ji and Kwon, 2017). Finally, the transcription factors ATF4 and CHOP are elevated upon PERK-Eif2α pathway activation and regulate numerous autophagy related genes (e.g., Atg5, Atg12, Atg16l1, LC3, and p62) and protein interactions (e.g., BCL-2 Beclin-1 binding) to promote autophagosome biogenesis and autophagy flux (B’Chir et al., 2013; Wang et al., 2014; Ji and Kwon, 2017; Zhu et al., 2010; Demishtein et al., 2017; McCullough et al., 2001; Kocaturk and Gozuacik, 2018).

In the N-end rule pathway, single N-terminal amino acids of proteins with positively charged and hydrophobic residues can act as N-degrons (Dougan et al., 2012). In the classical N-end rule pathway, an N-terminal Arg is recognized by proteins referred to as N-recognins. N-recognins such as the UBR Box family E3 ligases UBR1, UBR2, IBR4, and UBR5 ubiquitinate several substrates for degradation (Tasaki et al., 2013; Ji and Kwon, 2017). Additionally, the N-end rule pathway has been found to modulate autophagy clearance through p62 activity (Cha-Molstad et al., 2017). Under elevated stress conditions, such as upon proteasomal inhibition, an accumulation of misfolded and aggregated protein leads to relocalization of ER protein chaperones such as BiP. This cytosolic BiP is arginylated and binds to p62 causing a conformational change causing p62 to self-oligomerise and interact with LC3, thus promoting autophagosome targeting of proteins (Cha-Molstad et al., 2017; Figure 3). Further, p62 was reported to induce autophagosome biogenesis, increasing the number of autophagosomes as well as the amount of targeted protein sequestered within autophagosomes (Cha-Molstad et al., 2017).

**FIGURE 4:**
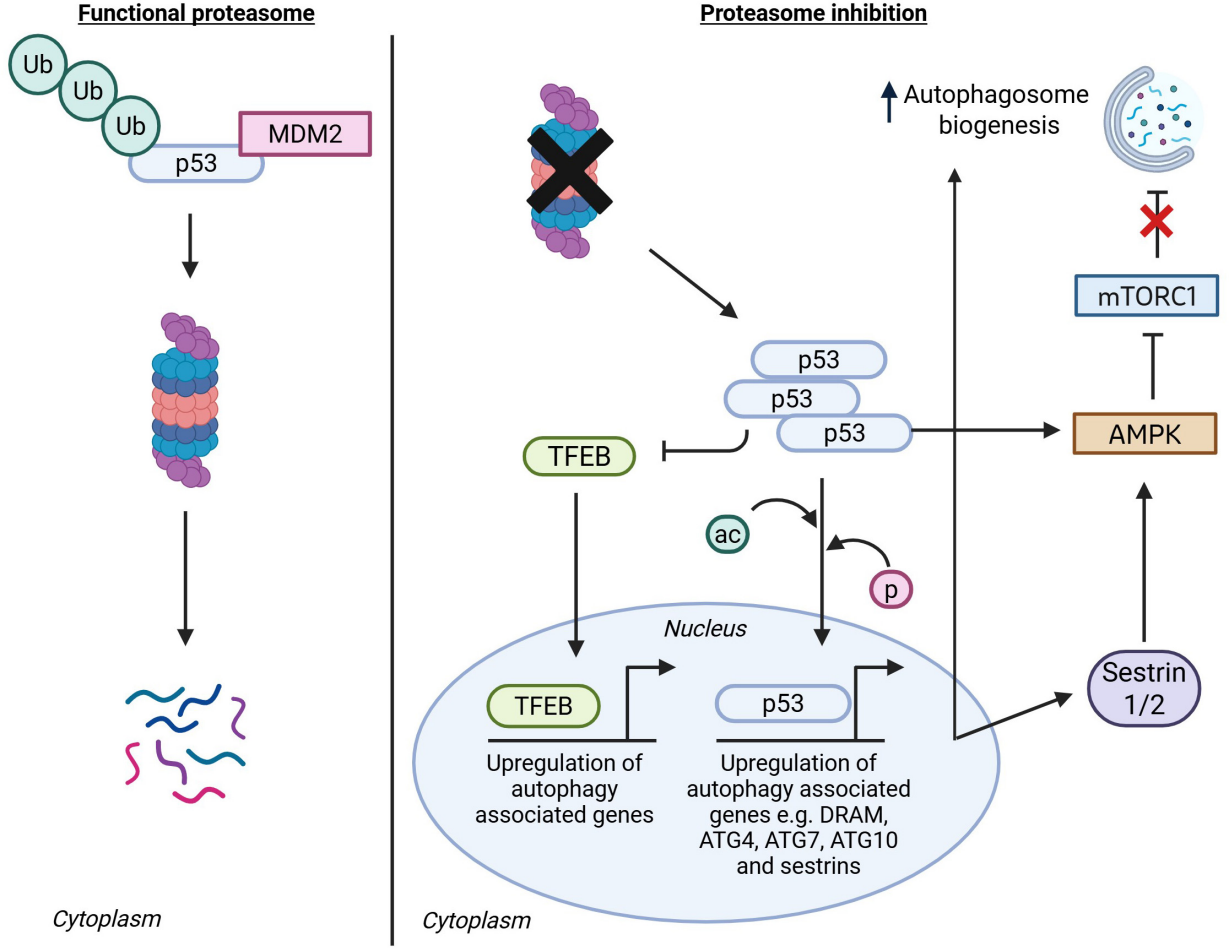
p53 in crosstalk from the UPS to autophagy. When the proteasome is functional, p53 degradation is facilitated by the E3 ligases like MDM2. When the proteasome is functionally impaired, p53 can be acetylated or phosphorylated by stress related kinases, which lead to p53 translocation into the nucleus where it upregulates the expression of autophagy associated genes and sestrin 1/2. Sestrin 1/2 and p53 can directly activate AMPK, leading to mTORC1 inhibition and autophagy activation. In a feedback mechanism, the accumulation of p53 can inhibit TFEB, a master transcriptional regulator of autophagy and lysosomal biogenesis. ac, acetylation; p, phosphorylation. Created in BioRender.com.

Transcriptional factor p53 has been shown to connect the UPS and autophagy through a number of pathways (Figure 4; Zhang et al., 2010). Under basal conditions, p53 levels are restricted by the activity of E3 ligases, e.g., MDM2 and subsequent degradation via the UPS, but levels can accumulate upon proteasome inhibition and cell stress (Lagunas-Martínez et al., 2017; Ji and Kwon, 2017; Du et al., 2009). In stress conditions, phosphorylated or acetylated p53 translocates to the nucleus, where it upregulates the expression of autophagy housekeeping genes such as damage-regulated autophagy modifier (DRAM), Atg2, Atg4, Atg7, and Atg10 (Crighton et al., 2006; Kocaturk and Gozuacik, 2018; Zhao G.-X. et al., 2015). Furthermore, p53 activates AMPK, both directly and through increased expression of activating sestrins, leading to mTOR inhibition and an increase in autophagy initiation (Zhang et al., 2010; Gülow et al., 2023). However, in contrast, elevated cytoplasmic p53 has also been shown to inhibit the nuclear translocation of TFEB, resulting in reduced expression of autophagy genes via a negative feedback loop (Zhang et al., 2017). This could represent a limiting autophagy induction feedback mechanism under prolonged stress or be driven by differences in cellular context (non-transformed vs. transformed), p53 localization, and mutational status.

In summary, inhibition of the UPS and the accompanying accumulation of toxic proteins is a signal for the induction of autophagy. That this signaling occurs through multiple different pathways is a testament to the importance of inducing autophagy to maintain cell survival during proteasomal stress. The outcome of these pathways focus on upregulating the expression of autophagy related genes to increase autophagosome biogenesis and increasing the number of vesicles available in the cell to degrade problematic proteins. However, what remains to be elucidated upon induction of autophagy through such pathways is how this is regulated over time, as excessive protein degradation can also result in cell death. An unimpaired UPS is responsible for the degradation of autophagy machinery and its regulators, presumably to keep the mechanism in check, but upon UPS disruption it would be important to understand how autophagy is kept in check after upregulation by the UPR and N-end rule or p53 pathways. Further, the basic canonical cell autonomous mechanisms of these pathways and their role of co-ordinating UPS to autophagy signaling are well established, however differences in temporal and spatial signaling in different neural cell types (e.g., neurons, microglia, astrocytes) is still elusive. A full deep dive into these cellular differences is beyond the scope of this review, however, having a greater understanding of this crosstalk between the UPS and autophagy in cell types that are differentially or commonly effected in neurodegenerative diseases will be crucial when trying to harness these mechanisms as a therapeutic treatment.

## Regulation of the UPS by autophagy

**FIGURE 5:**
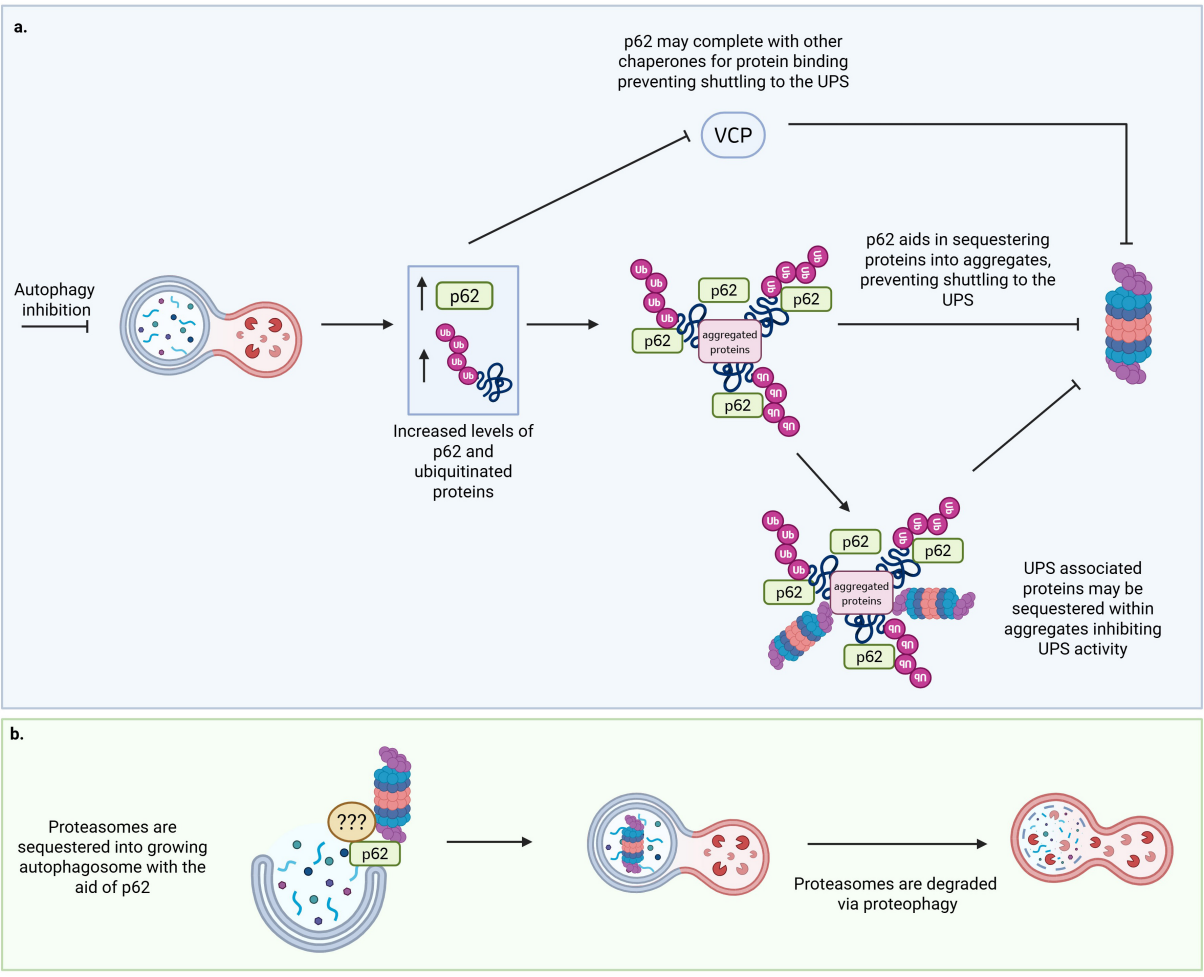
Cross talk from autophagy to the UPS. **(a)** Autophagy inhibition leads to a reduction in UPS substrate flux. Increases in the levels of p62 compete with other chaperones to bind to proteins and sequester aggregates. These p62 protein-aggregate complexes delay the delivery of proteins to the UPS, and may sequester proteasomal subunits and associated proteins, leading to a reduction in UPS activity. **(b)** Increased autophagy activity may increase the degradation of proteosomes via proteaphagy, with the aid of p62. Created in BioRender.com.

Whilst there is compelling evidence to support that inhibition of the UPS triggers a compensatory increase in autophagy, inhibition of autophagy has been reported to both increase and decrease proteasome activity through different mechanisms. Korolchuck et al., reported that inhibition of autophagy lead to the accumulation of p62 and protein aggregates. Elevated levels of p62 increases competition for substrate binding with other chaperone proteins, e.g., VCP, which delays protein shuttling to the UPS (Korolchuk et al., 2009). Protein aggregates complexed with p62 may also sequester proteasomal subunits or UPS associated proteins, reducing overall UPS activity (Korolchuk et al., 2010; Figure 5a). In contrast, Wang et al reports that impairment of autophagy leads to an increase in the levels of proteasome subunit β5, which correlated to increased chymotrypsin-like activity (Wang et al., 2013). This data suggests that a dysfunction in autophagy may preserve or increase proteasome activity, but ultimately the substrate flux is likely impaired via sequestration of proteasome substrates via p62.

The degradation of dysfunctional proteasomes via the autophagy pathway is a phenomenon termed “proteaphagy” (Cohen-Kaplan et al., 2016b,Bartel, 2015; Goebel et al., 2020). In yeast, proteaphagy has been reported to be dependent on selective autophagy receptor Atg11 as well as multiple MAP kinases (Waite et al., 2022), whilst in Arabidopsis, proteaphagy is mediated by Rpn10 (Richard et al., 2015). In mammalian cells the mechanisms of proteaphagy are less clear (Goebel et al., 2020). Experiments that utilized autophagy activators (e.g., pan-FGFR inhibitor LY2874455) suppressed the expression of pro-inflammatory factors by increasing the autophagic degradation of immunoproteasomes that cleave factors for antigen presentation (Zhou et al., 2024). Stress conditions (e.g., amino acid starvation) can dramatically change the ubiquitination signature of the 26S/30S proteasome by increasing the ubiquitination of Rpn2, Rpn10, and Rpn13, and reducing the ubiquitination of Rpn5/6 and α7 (Cohen-Kaplan et al., 2016b). Upon stress induction different sites are differentially ubiquitinated on the proteasome subunit Rpn1, whilst UPS associated proteins such as E3 ligases HUWE1 and KCMF1 are also ubiquitinated (Cohen-Kaplan et al., 2016b). Interestingly, the co-localisation of autophagy and proteasome markers in membrane bound structures has led to the proposal of a distinct degradative compartment containing active proteasomes to enhance the digestion of contents termed the ‘autophagoproteasome’ (Pasquali et al., 2009). These structures have been shown to be heavily mTOR activity dependent and exist at the nexus of both degradation pathways, suggesting a potential role as an integrated proteostasis hub to enhance protein clearance (Lenzi et al., 2016; Limanaqi et al., 2021). Future work clarifying the exact functional role of these structures may yield interesting new regulators of both pathways.

Choi and colleagues demonstrated that CHIP (an E3 ligase) is essential for ubiquitylation of defective proteasomes, and that proteasomes are shuttled to aggresome formation sites by histone deacetylase 6 (HDAC6) and dynein mediated transport (Choi et al., 2020; Fusco et al., 2012). Several studies implicate p62 in proteasomal sequestration into aggregates and in linking proteasomes to autophagy via autophagy receptor LC3 (Choi et al., 2020; Cohen-Kaplan et al., 2016a; Figure 5b). The biology of p62 positive aggresomes and proteaphagy is complex, whilst aggresome formation may function to sequester defective proteasomes and associated proteins together for autophagic degradation, when proteasomes are functional, p62 condensates have been reported to be a hub for proteasome mediated turnover (Erdbrügger and Wilfling, 2021).

There is less clarity in the literature when it comes to autophagy’s role in regulating the UPS, but the role of proteaphagy in maintaining proteostasis is likely very important, with the removal of inactive proteasomes giving way for the activities of functional units. However, little is known about the proteins that regulate the selectivity of proteasome to autophagosomes for degradation. Fully elucidating this phenomenon will be important for the progression of this field.

## UPS and autophagy pathways share molecular determinants and substrates

Though the UPS and autophagy degrade proteins through distinct mechanisms, it is evident that the two systems share common substrates and molecular determinants. Examples of proteins that link the two systems include ubiquitin chain signaling, transcription factors, E3 ligases, DUBs, and chaperone proteins. The existence of multiple shared entities between the two systems reflects the importance of communication and cross-regulation to maintain proteostasis. Understanding the biology underpinning proteins that regulate both systems may be paramount in drug discovery, especially for conditions where disease progression is linked to protein accumulation, i.e., AD, ALS/FTD, PD and HD. Table 1 summarizes proteins that regulate both the UPS and autophagy and describes their involvement in neurodegenerative disease pathology.

**TABLE 1:** Summary of proteins that regulate UPS and autophagy pathways and their implications in neurodegenerative disease.

Gene symbol	Protein	Functions in UPS and autophagy	Implications in neurodegenerative diseases	References
*ATF4*	ATF4	Signaling from UPS to autophagy: Transcription factor upregulated in the Perk-Eif2a branch of the UPR. Leads to upregulation of autophagy genes.	Dysregulation of ATF4 levels observed in AD, PD and HD. ATF4 activates pro-survival and pro-death signaling during neurodegeneration.	(B’Chir et al., 2013; Pitale et al., 2017)
*ATF6*	ATF6	Signaling from the UPS to autophagy: Transcription factor in the ATF6 branch of UPR, leads to increase in autophagy by upregulation of DAPK1, which phosphorylates Beclin-1 for autophagosome formation.	UPR via ATF6 declines during ALS progression. ATF6 reduced. ATF6 expression is reduced in APP/PS1 mouse models of AD.	(Gade et al., 2012; Prell et al., 2019; Zalckvar et al., 2009; Du et al., 2020)
*BAG1/BAG3*	BAG1/BAG3	BAG1 and BAG3 compete for HSP70 biding to degrade target proteins by either the UPS or autophagy, respectively.	The vulnerability of excitatory neurons to develop tau pathology correlates with a reduced expression of BAG3 compared to less vulnerable inhibitory neurons. BAG3 promotes the clearance of tau, alpha-synuclein, mutant SOD1 and mutant huntingtin in models of neurodegeneration.	(Carra et al., 2008b; Gamerdinger et al., 2009; Lei et al., 2015; Liebl and Hoppe, 2016; Cao et al., 2017; Fu et al., 2019; Lin et al., 2023)
*BCL2*	BCL-2	Protein bound to Beclin-1 in non-starvation conditions to inhibit autophagy. UPS to autophagy signaling can regulate BCL-2 activity through phosphorylation via p-JNK as an end result of the IRE1-TRAF2 UPR branch.	BCL-2 levels decrease in SOD1 overexpression mouse model of ALS. Overexpression of Bcl-2 in SOD1 transgenic mice results in delayed onset of symptoms and increased survival.	(Mu et al., 1996; Kostic et al., 1997; Akhtar et al., 2004; Deegan et al., 2013; Ji and Kwon, 2017; Wei et al., 2008)
*CRIP1*	CRIP1	Protein reported to regulate both UPS and autophagy. Stabilizes PA200.	Possible use as a CAA biomarker in AD and interaction with AB and modulation of inflammatory environment	(Tang et al., 2024; Wojtas et al., 2025)
*Cul4A*	Cullin-4	E3 Ligase, ubiquitinate targets for degradation via both the UPS and autophagy	Implicated in UPS and autophagy pathways, which are disrupted in neurodegenerative diseases	(Antonioli et al., 2014)
*DDIT3*	CHOP	Signaling from UPS to autophagy: PERK-Eif2A UPR pathway, downregulates BCL2 binding to Beclin-1, increasing autophagy	Elevated protein expression in AD, PD and ALS likely driven by increased UPR stress	(McCullough et al., 2001; Kocaturk and Gozuacik, 2018; Lee J. H. et al., 2010; Gegg et al., 2012; Ito et al., 2009)
*FOXO3*	FOXO3	Transcription factor that controls the expression of proteasome and autophagy genes.	FOXO3 has a proposed dual role in AD, PD and HD, being neuroprotective in early stages but neurotoxic at later stages with prolonged activation.	(Zhao et al., 2007; Lilienbaum, 2013; Shi C. et al., 2016; Schäffner et al., 2018; Du and Zheng, 2021)
*NFKB*	NF-κB	Transcription factor that controls expression of numerous proteasome and autophagy genes	NF-κB target genes are also involved in cell stress and apoptosis, which is especially prominent in neurodegenerative diseases. NF- Kb inhibition explored as a treatment for neurodegeneration.	(Sanchez-Lopez et al., 2020; Ossendorp et al., 2005; Vadlamudi and Shin, 1998; Singh and Singh, 2020)
*NRF1*	NRF1	Transcription factor that controls expression of numerous proteasome and autophagy machinery genes, and associated proteins.	PD patients display reduced levels of NRF1 in dopaminergic neurons of the substantia nigra. NRF1 deletion in mice induces neurodegeneration phenotypes.	(Lee C. S. et al., 2011 Hamazaki and Murata, 2020; Villaescusa et al., 2016; Sedlacek et al., 2025)
*NRF2*	NRF2	Transcription factor that controls expression of numerous proteasome and autophagy machinery genes, and associated proteins.	In neurodegenerative diseases, NRF2 may be suppressed or activated depending on the cell type and disease stage. NRF2 is heavily implicated in transcriptional regulation of pathways associated with oxidative stress, inflammation and mitochondrial dysfunction, which are also implicated in ageing and neurodegenerative diseases.	(Pajares et al., 2017; Pickering et al., 2012; Sun-Wang et al., 2020; Dodson et al., 2019; Brandes and Gray, 2020)
*PRKN*	Parkin	E3 ligase ubiquitinates mitochondria for removal via mitophagy and recruits proteasomes to the mitochondria for degradation of proteins on the outer mitochondria membrane	A number of mutations in Parkin have been identified as causing PD, these mutations impair mitochondrial ubiquitination leading to reduced mitophagy.	(Chan et al., 2011; Lee J. Y. et al., 2010)
*SQSTM1*	P62	Chaperone protein that can direct proteins to the UPS or autophagy for degradation, also plays important roles in sequestration of proteins into aggresomes.	Mutation of SQSTM1 cause ALS/FTD. Aggregation of p62 with other pathological proteins observed in AD, PD, ALS, FTD, HD and PD.	(Wurzer et al., 2015; Pohl and Dikic, 2019; Liu W. J. et al., 2016; Ma et al., 2019)
*STUB1*	CHIP	CHIP is a molecular chaperone that can bind Hsp70/90 via its TPR domain to target proteins for proteasomal degradation. The U-box domain of CHIP has been reported to ubiquitinate proteins (E3 ligase activities) and targeting proteins for autophagy degradation via its U-Box domain.	Mutations in CHIP cause spinocerebellar Ataxia, a neurodegenerative disorder. CHIP can promote the degradation of amyloid-beta, tau, mutant SOD1, mutant huntingtin and alpha-synuclein in neurodegenerative models.	(Edkins, 2015; Demand et al., 2001; Dickey et al., 2006; Shin et al., 2005; Zhou et al., 2014; Zhang S. et al., 2020)
*TP53*	p53	Signaling from UPS to autophagy: Transcription factor that controls the expression of a number of autophagy housekeeping genes.	P53 is activated in cell stress and promotes cell death in AD, PD, ALS and HD.	(Crighton et al., 2006; Kocaturk and Gozuacik, 2018; Luo et al., 2022; Szybińska and Leśniak, 2017)
*TRIM16*	TRIM16	Stabilizes Nrf2 leading to an upregulation of autophagy and proteasome gene expression. Also acts as a scaffolding protein aiding in autophagy.	Implicated in UPS and autophagy pathways, which are disrupted in neurodegenerative diseases	(Jena et al., 2018a,Jena et al., 2018b)
*TRIM44*	TRIM44	Involved in cross-talk between UPS and autophagy. After proteasome inhibition TRIM44 bough k48-ubiquitinated targets and delivered them to autophagy pathway and promoted p62 oligomerization.	Implicated in UPS and autophagy pathways, which are disrupted in neurodegenerative diseases	(Lyu et al., 2022)
*TRIM50*	TRIM50	Involved in cross-talk between UPS and autophagy. After proteasome inhibition TRIM50 promotes autophagy by through interactions with p62 and HDAC6.	Implicated in UPS and autophagy pathways, which are disrupted in neurodegenerative diseases	(Fusco et al., 2012)
*UBB*	Ubiquitin	Small protein that signals for protein degradation via the UPS or autophagy by binding target proteins in a range of unbranched and branched conformations	Ubiquitin decorates pathological aggregates in AD, PD, FTD/ALS, and HD.	(Liu W. J. et al., 2016; Dwane et al., 2017; Riley et al., 2010; Feng et al., 2017)
*UCHL5*	UCHL5	DUB reported to be involved in regulation of both UPS and autophagy signaling	Implicated in UPS and autophagy pathways, which are disrupted in neurodegenerative diseases	(Jha et al., 2025)
*USP14*	USP14	DUB reported to be involved in regulation of both UPS and autophagy signaling	Implicated in UPS and autophagy pathways, which are disrupted in neurodegenerative diseases	(Lee et al., 2016; Lee J. Y. et al., 2010; Lee M. J. et al., 2011; Kim et al., 2018)
*VCP*	VCP	Chaperone/AAA-ATPase enzyme implicated in multiple pathways across the UPS and autophagy	Mutations in VCP cause ALS/FTD and multisystem proteinopathies.	(Ferrari et al., 2022; Meyer and Weihl, 2014; Johnson et al., 2010)
*XBP1*	XBP1s	Signaling form UPS to autophagy: IRE1-TRAF2 branch of the UPR, transcription factors that controls expression of core autophagy genes	Increased spliced XBP1 found in AD, human models of PD, reduced in TDP-43 ALS models, and depletion shown to be neuroprotective in SOD1 ALS models	(Margariti et al., 2013; Lee J. Y. et al., 2010; Heman-Ackah et al., 2017; Tong et al., 2012; Hetz et al., 2009)

## Transcription factors/regulators

**FIGURE 6:**
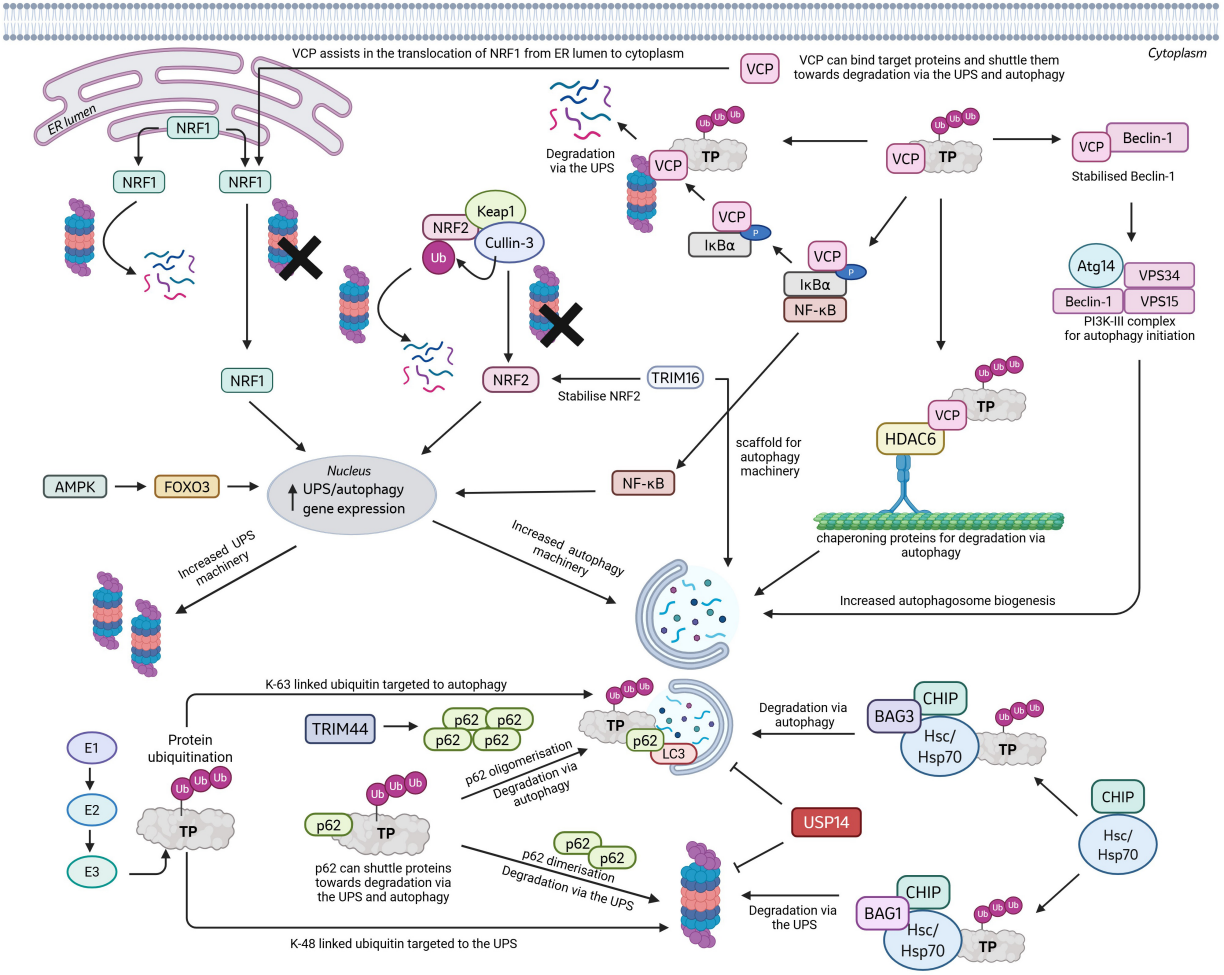
The stabilisation and activation of transcription factors NRF1, NRF2, FOXO3 and NF-κB leads to the upregulation of both UPS and autophagy associated genes required to boost the level of UPS and autophagy machinery. NRF1 is translocated from the ER lumen to the cytoplasm with the aid of VCP and is regulated by proteasomal degradation. Cytoplasmic NRF2 is also regulated by proteasomal degradation with the assistance of Keap1 and Cullin-3. TRIM16 helps stabilise NRF2, whilst also acting as a scaffold for autophagy machinery. FOXO3 is activated by AMPK. VCP regulates NF-κB by binding IκBα and targeting it for degradation via the proteasome, freeing NF-?B for its transcriptional activities. VCP can bind to target proteins and chaperone them to both the UPS and autophagy for degradation. VCP mediated autophagy degradation involves binding HDAC6 for transportation to the site of phagophore generation as well as stabilising Beclin-1 to allow for its integration into the PI3K complex. Through an E1/E2/E3 mediated mechanism, target proteins are signalled for degradation by ubiquitination. Typically, K48-linked ubiquitination targets proteins for degradation via the UPS, whereas K63 ubiquitination targets proteins to degradation via autophagy. p62 can shuttle proteins towards the UPS or autophagy for degradation depending on its oligomerisation stage with p62 dimerisation targeting proteins to the UPS, and oligomerisation steering targets towards degradation via autophagy. USP14 can inhibit protein degradation via the UPS and autophagy by cleaving ubiquitin pre-maturely. Finally, the CHIP/Hsp70 complex, when bound to BAG3, chaperones proteins towards autophagy, whereas binding to BAG1 chaperones proteins towards the UPS. Created in BioRender.com.

The transcription factors Nuclear factor erythroid-derived 2 related factors 1 and 2 (NRF1 and NRF2) have been reported to be important in linking the UPS and autophagy systems. NRF1 predominantly resides in the ER lumen (Hamazaki and Murata, 2020), whereas NRF2 is located in the cytoplasm. Under normal conditions NRF1 levels are kept in check by ER associated degradation (ERAD) via the proteasome. However, during proteasome inhibition, NRF1 is retro-translocated into the nucleus, where it induces the transcription of a wide range of core and regulatory proteasome subunits to increase functional proteasome expression and counteract inhibition (Figure 6; Hamazaki and Murata, 2020; Radhakrishnan et al., 2010; Lee C. S. et al., 2011). Further, NRF1 induces the expression of autophagy related genes p62 and GABARAPL1 (Sha et al., 2018; Hatanaka et al., 2023). Interestingly, PD patients display reduced levels of NRF1 in dopaminergic neurons of the substantia nigra (Villaescusa et al., 2016; Sedlacek et al., 2025), and research demonstrates that NRF1 deletion in mice induces neurodegeneration phenotypes (Lee C. S. et al., 2011), making NRF1 activation an interesting therapeutic target. Cytoplasmic NRF2 in non-stress conditions is bound to Keap1, which acts as a substrate adapter for E3 ligase Cullin-3 (McMahon et al., 2003; Zhang et al., 2004). In turn, Cullin-3 ubiquitinates NRF2 to promote its degradation via the UPS. During proteasome inhibition, NRF2 dissociates from Keap1 and upon translocation to the nucleus, increases the transcription of proteasome and autophagy subunits, including p62 (Figure 6; Sun-Wang et al., 2020; Pajares et al., 2017; Liu W. J. et al., 2016, Jang et al., 2014; Pickering et al., 2012). In neurodegenerative diseases, NRF2 may be suppressed or activated depending on the cell type and disease stage, for example, elevated nuclear localization of NRF2 is reported in the substantia nigra of PD patients, whereas NRF2 levels are reduced in spinal cord and primary motor cortex of ALS patients (Dodson et al., 2019; Sarlette et al., 2008). Further, NRF2 expression is reported to be reduced in age associated reduction of neural cell activity (Darabi et al., 2018; Corenblum et al., 2016). As well as regulating UPS and autophagy, NRF2 is heavily implicated in transcriptional regulation of pathways associated with oxidative stress, inflammation and mitochondrial dysfunction which are also implicated in aging and neurodegenerative diseases, making NRF2 a target of interest for drug discovery (Brandes and Gray, 2020).

The transcription factor FOXO3 has been implicated in stimulating overall protein degradation via both the UPS and autophagy (Zhao et al., 2007; Lilienbaum, 2013; Schäffner et al., 2018). Under high stress conditions, AMPK can activate FOXO3 through phosphorylation at S413 and S588 (Singh A. et al., 2021; Davila et al., 2012). FOXO3 activation leads to transcription of numerous autophagy related genes including Atg4B, Atg12, Beclin-1, Bnip3, GABARAPL1, LC3B, ULK2, and Vsp34 (Zhao et al., 2007), as well as E3 ligases important for protein degradation via the UPS (e.g., muscle specific E3 ligases MuRF-1 and MAFbx and atrogin-1) (Figure 6; Rom and Reznick, 2016; Sandri et al., 2004), In turn, these FOXO3 levels are regulated via MDM2 ubiquitination and degradation by the UPS (Fu et al., 2009). Dopaminergic neuron health is reported to be particularly susceptible to changes in FOXO3. Indeed, constitutive activation of FOXO3 in mice leads to apoptotic signaling and cell death in the substantia nigra, however, lower levels of FOXO3 activation reduced the levels of pathological alpha-synuclein in PD models via increased autophagy and increased protein clearance (Pino et al., 2014). Therefore, FOXO3 levels would require precise modulation to avoid the safety concerns associated with high levels of expression. Despite this, the ability of FOXO3 to modulate protein degradation in this way has been identified to contribute healthy aging and human longevity making this an interesting target for further investigation (Morris et al., 2015; Hu et al., 2019).

The transcription factor family NF-κB (Nuclear factor kappa-light-chain-enhancer or activated B cells) regulates the transcription of an extensive list of genes, including those linked to both the UPS and autophagy, e.g., Proteasome activator complex subunit 2, p62, and Beclin-1 (Figure 6; Sanchez-Lopez et al., 2020; Ossendorp et al., 2005; Vadlamudi and Shin, 1998; Bhatnagar et al., 2012; Singh A. et al., 2021). Unfortunately, many NF-κB target genes are also involved in cell stress, neuroinflammation and apoptosis, which are especially prominent in neurodegenerative diseases and therefore inhibition rather than activation of NF-κB has been explored as a potential treatment (Singh and Singh, 2020).

## Adapter proteins, chaperones, E3 ligases and DUBS

The capacity of p62 to chaperone ubiquitinated substrates to both the proteasome and the growing autophagosome for degradation, implicates this receptor in numerous pathways connecting the two systems (Liu W. J. et al., 2016). p62 binds ubiquitin via its UBA domain, to the proteasome via its Phox and Bem1p region (PB1), and to autophagosome membranes via its LC3-interacting region (LIR) (Figure 6; Rogov et al., 2014; Pohl and Dikic, 2019). The pathway choice of p62 is determined by its oligomeric state (Lu et al., 2017; Pohl and Dikic, 2019). For example, p62 dimers bind K48-linked proteins via the UBA domain and shuttles them to the proteasome for degradation, whereas K63-linked aggregated proteins are sequestered by oligomeric p62 for degradation via autophagy (Pohl and Dikic, 2019; Wurzer et al., 2015). Importantly, as a positive feedback loop, p62 also binds via its KIR motif to Keap1, shuttling the protein toward autophagic degradation. This disruption in the Keap1-NRF2 complex leads to increased free NRF2, which translocates to the nucleus and upregulates the transcription of many UPS and autophagy associated genes, including p62, resulting in a positive feedback loop (Hennig et al., 2021). However, Cul3, the E3 ligase bound to Keap1 can ubiquitinate p62 at K420, leading to its degradation (Lee et al., 2017). The affinity of p62 to bind Keap1 is strongly increased by p62 phosphorylation at S349 (Hennig et al., 2021; Ichimura et al., 2013). When amino acids are abundant, MEKK3 (mitogen-activated protein kinase kinase 3) phosphorylates p62 at T269 and S272. Following phosphorylation, p62 binds the E3 ligase TRAF6 (via its TRAF6-binding domain) and raptor (a subunit of mTORC1), inducing K63-linked mTOR ubiquitination which activates mTORC1, thereby supressing autophagy induction (Hennig et al., 2021). Further, as discussed above, p62 is involved in the activation and stabilization of transcription factor NF-κB which regulates the transcription of UPS and autophagy genes, including p62 itself (Hennig et al., 2021).

Valosin-containing protein (VCP or p97) is an AAA-ATPase chaperone protein that plays an important role in both UPS and autophagy (Figure 6; Ferrari et al., 2022). VCP works to unfold proteins that are tightly folded or embedded within larger multiprotein complexes/membrane structures to allow more efficient targeting and delivery to the proteasome (Meyer and Weihl, 2014). Further, VCP is implicated in multiple steps of autophagy, including transcriptional regulation of autophagy genes through regulation of TFEB and NF-κβ (Arhzaouy et al., 2019; Asai et al., 2002), regulating autophagy initiation by stabilizing Beclin-1 and enhancing initiation complex formation (Hill et al., 2021) and routing substrates to autophagic degradation with the assistance of HDAC6 (Boyault et al., 2006). VCP is also crucial for the translocation of NRF1 from the ER into the cytoplasm, meaning it has an upstream effect in regulating proteasome and autophagy gene expression (Radhakrishnan et al., 2014). Of relevance, over 50 mutations in VCP have been identified to cause familial ALS/FTD and multisystem proteinopathy. Indeed,10% of individuals with mutated VCP develop ALS, accounting for 1–2% of all familial ALS cases, while around 30% of VCP mutant cases develop FTD, accounting for 3.5% of total FTD cases, and importantly, both diseases on this spectrum are characterized by the accumulation of pathological FUS and TDP-43 (Johnson et al., 2010). Overexpression of VCP mutants *in vitro* (R95G, R155H, R155C, R191Q, and A232E) have been shown to decrease proteasome activity and induce ER stress and cell death (Gitcho et al., 2009), and genetic KO or inhibition significantly increases *in vitro* pathological seeding of alpha-synuclein and TDP-43 (Zhu et al., 2022). Collectively, these data implicate VCP heavily in both proteasome and autophagy regulation and the development of neurodegenerative proteinopathies.

The E3 ligase Parkin targets dysfunctional mitochondria for degradation via a selective autophagy process known as mitophagy (Narendra and Youle, 2024). Parkin can act as a surveillance protein, monitoring the health of the mitochondria population within a cell. Upon identification of dysfunctional mitochondria, Parkin, in concert with PINK1, marks the mitochondria for autophagic degradation via K63-linked ubiquitination (Geisler et al., 2010; Matsuda et al., 2010; Narendra et al., 2008; Narendra et al., 2010; Vives-Bauza et al., 2010). A number of mutations in Parkin have been identified as causing PD, these mutations impair mitochondrial ubiquitination leading to reduced mitophagy (Lee J. Y. et al., 2010). Parkin is also involved in the recruitment of 26S proteasomes to the mitochondria to facilitate the degradation of mitochondrial outer membrane proteins, as a prerequisite for mitophagy (Chan et al., 2011). Parkin can directly activate the 26S proteasomes *in vitro* and enhances the formation of 19s RP, participating in the assembly and maintenance of the proteasome (Um et al., 2010). Further, Parkin KO reduces proteasome activity in mice and Drosophila, and several PD associated parkin mutations (e.g., R42P and T240R) are reported to induce proteasome dysfunction in cell lines (Kyratzi et al., 2007).

Numerous members of the Tripartite motif containing protein (TRIM) family have been reported to aid in the degradation of proteins via the UPS (e.g., TRIM5/8/11/19/21/22/36), autophagy (e.g., TRIM5) or both systems (e.g., TRIM16/50/44). TRIM16 is involved in increasing the turnover of misfolded proteins by stabilizing NRF2, to upregulate both UPS and autophagy genes (Jena et al., 2018b,Zhang L. et al., 2020; Jena et al., 2018a) and can act as a scaffold protein during autophagy by interacting directly with p62, LC3B, ULK1, and ATG16L (Jena et al., 2019; Jena et al., 2018b,Jena et al., 2018a). The E3-ligase TRIM50 interacts with HDAC6 and p62 to promote the clearance of ubiquitinated proteins via autophagy when the proteasome is impaired (Fusco et al., 2012). Elevated levels of ubiquitinated TRIM44 after suppression of UPS activity can upregulate autophagy by promoting p62 oligomerization (Lyu et al., 2022). TRIM21 has a novel mechanism of action whereby it recognizes the Fc region of antibody bound target proteins and requires the clustering of the RING domain to activate the E3 ligase activity. This allows for selective degradation of the aggregated species of proteins whilst sparing the monomeric form, thus making this an interesting novel approach for clearing aggregate prone proteins (e.g., Tau, Huntingtin, etc.) in neurodegenerative disease (Zeng et al., 2021; Benn et al., 2024). TRIM21 can also modulate degradation of substrates via autophagy by ubiquitinating substrates such as cMyc (Ye et al., 2024), G3BP (Yang et al., 2023), IRF3 (Kimura et al., 2015), and RBM38c (Xia et al., 2025) via K63 linkage. In addition, TRIM21 can increase autophagosome biogenesis by selectively degrading the NCAPH mediated activation of the AKT/mTOR pathway in multiple myeloma cell lines (Wang et al., 2024).

C-terminal Hsp70 binding protein (CHIP) is a molecular chaperone/E3 ligase demonstrated to determine protein degradation fate by either the UPS or autophagy (Figure 6). CHIP can bind heat shock protein Hsp70/90 via its tetratricopeptide repeat (TPR) domain, which in turn can bind a multitude of unfolded proteins targeted for degradation (Edkins, 2015). BAG chaperone 1 (BAG1) interacts with Hsp70 via its BAG domain and then chaperones this CHIP/Hsp70 complex to the proteasome for target degradation (Demand et al., 2001). Whilst aiding in shuttling proteins toward proteasomes, CHIP can also function as an E3 ligase through its RING-finger (U-Box) domain, to ubiquitinate target proteins for degradation via the proteasome (Edkins, 2015; Demand et al., 2001; Dickey et al., 2006) and autophagy (Kocaturk and Gozuacik, 2018; Shin et al., 2005; Zhou et al., 2014).

Whilst BAG1 has been demonstrated to be important for chaperoning proteins to the UPS for degradation, BAG3 is important for sequestering proteins during proteasome inhibition and redirecting them to degradation via autophagy in what is often described as the “proteasome-to-autophagy switch” (Liu et al., 2013; Minoia et al., 2014). During this switch, BAG3 competes with BAG1 for binding to Hsp70 (Liebl and Hoppe, 2016) and forms a complex with HspB8. Within this complex, HspB8 is responsible for recognizing misfolded proteins, whilst BAG3 recruits and activates the autophagy machinery (Carra et al., 2008a). It is unclear what factors determines the BAG1-BAG3 switch decision, but BAG1 and 3 are regulated by distinct transcription factors; Sp1 controlling BAG1 expression and HSF1, WT1, and Egr1 controlling BAG3 expression. Further, the BAG1 pathway predominates under normal conditions, whilst the switch to BAG3 increases under cell stress and during age (Stürner and Behl, 2017; Townsend et al., 2003; Marzullo et al., 2020). Using weighted gene co-expression network analysis, BAG3 has been reported to operate as a master regulator gene, as the vulnerability of excitatory neurons to develop tau pathology correlated with a reduced expression of BAG3 compared to less vulnerable inhibitory neurons (Fu et al., 2019). These observations make BAG3 an interesting target for rescuing protein homeostasis in neurodegenerative diseases. Another more recent chaperone-based mechanism also describes how the proteasome and autophagy systems act synergistically to dissaggregate pathogenic huntingtin aggregates via the HSP70-HSP110-DNAJB6 complex and 19S proteasome system. This system acts to fragment and compact aggregates to induce clustering of autophagy receptors and process aggregate clearance by autophagy (aggrephagy) (Mauthe et al., 2025).

USP14, a proteasome associated DUB, has been reported to regulate the UPS and autophagy in many complex and conflicting ways. The most well understood primary mechanism of action is to assist in protein degradation management by binding to the proteasome and deubiquitinating targeting substrates before their commitment to degradation (Lee M. J. et al., 2011). This deubiquitination of targeted substrates occurs via the C-terminal catalytic USP domain of USP14 (Lee et al., 2016), and the pre-mature ubiquitin chain trimming of targeted proteins (e.g., tau) by USP14, has been reported to prevent target degradation (Boselli et al., 2017; Hu et al., 2005). In addition to its catalytic role, USP14 can also modulate proteasome activity through non-catalytic mechanisms, such as via its interaction with the Rpn13 subunit. This has been shown to restrict the conformational changes required for substrate translocation into the core particle, thereby suppressing proteolysis (Kim et al., 2018; Bashore et al., 2015). In contrast, interaction of the USP14 UBL domain with proteasomal subunit Rpn1 mimics the effect of ubiquitin binding to the proteasome, leading to conformational changes within the CP and increasing protein degradation (Kim and Goldberg, 2018). Together these data suggest that USP14 may exert bidirectional catalytic and non-catalytic control over proteasome activity in a context dependent manner and is driven by either proteasomal engagement or ubiquitin occupancy.

The role of USP14 in autophagy regulation is less well defined and supported by largely variable cell-based studies. One study demonstrated a reduction in autophagy upon USP14 inhibition, which has largely been attributed to increased proteasomal degradation of the autophagy maturation regulator UVRAG (Figure 2; Kim et al., 2018). Similarly, Jha et al., reported that downregulation of UCHL5 (another proteasome associated DUB) and USP14 via siRNA, suppressed autophagy flux in HeLa cells by blocking autophagosome-lysosome fusion (Jha et al., 2025). In contrast, Xu et al showed that USP14 inhibition prevents trimming of K63 linked ubiquitin chains on Beclin1, and thereby decreasing Beclin1 affinity for Atg14L/UVRAG to promote Atg14L/UVRAG complex formation and increase autophagy flux (Xu et al., 2016). These data suggests that USP14 can influence autophagy both directly and indirectly, with net outcomes determined by the temporal dynamics of UPS modulation and the stability of key autophagy factors.

Recently, CRIP1 (Cystein-rich protein 1) a member of the LIM/double zinc finger protein family, was reported to dually regulate the activities of both the UPS and autophagy in multiple myeloma cell lines through simultaneously binding the DUB USP7 and the proteasome activator PA200. The stabilization of PA200 appears to be an important factor for the increased activity of both UPS and autophagy, but how this stabilization relates to autophagy activity remains unclear (Tang et al., 2024).

The stabilization and activation of transcription factors NRF1, NRF2, FOXO3 and NF-κB leads to the upregulation of both UPS and autophagy associated genes required to boost the level of UPS and autophagy machinery. NRF1 is translocated from the ER lumen to the cytoplasm with the aid of VCP and is regulated by proteasomal degradation. Cytoplasmic NRF2 is also regulated by proteasomal degradation with the assistance of Keap1 and Cullin-3. TRIM16 helps stabilize NRF2, whilst also acting as a scaffold for autophagy machinery. FOXO3 is activated by AMPK. VCP regulates NF-κB by binding IκBα and targeting it for degradation via the proteasome, freeing NF-κB for its transcriptional activities. VCP can bind to target proteins and chaperone them to both the UPS and autophagy for degradation. VCP mediated autophagy degradation involves binding HDAC6 for transportation to the site of phagophore generation as well as stabilizing Beclin-1 to allow for its integration into the PI3K complex. Through an E1/E2/E3 mediated mechanism, target proteins are signaled for degradation by ubiquitination. Typically, K48-linked ubiquitination targets proteins for degradation via the UPS, whereas K63 ubiquitination targets proteins to degradation via autophagy. p62 can shuttle proteins toward the UPS or autophagy for degradation depending on its oligomerization stage with p62 dimerization targeting proteins to the UPS, and oligomerization steering targets toward degradation via autophagy. USP14 can inhibit protein degradation via the UPS and autophagy by cleaving ubiquitin pre-maturely. Finally, the CHIP/Hsp70 complex, when bound to BAG3, chaperones proteins toward autophagy, whereas binding to BAG1 chaperones proteins toward the UPS.

## Small molecule dual activation of the UPS and autophagy as a therapeutic for neurodegenerative diseases

As previously discussed, numerous transcription factors, protein chaperones, E3 ligases and DUB proteins have been implicated in modulating the activities of both the UPS and autophagy, and several of these identified regulators are promising targets for new therapeutics in neurodegenerative diseases.

SMER28 is a small molecule that binds to VCP to selectively increase its D1 ATPase activity. Wrobel and colleges elegantly demonstrated that through binding VCP, SMER28 elevates autophagosome biogenesis by enhancing interactions between proteins in the PI3K complex, resulting in increased PI3P production and accelerating VCP dependent proteasomal clearance of aggregation prone substrates (Wrobel et al., 2022). Indeed, SMER compounds have demonstrated preclinical efficacy as mTOR independent autophagy enhancers that attenuate mutant huntingtin-fragment toxicity *in vitro* and *in vivo* (Sarkar et al., 2007), downregulate Aβ40/ Aβ42 and APP-CTF levels in cellular models of Alzheimer’s disease (Tian et al., 2011) and attenuate 6-hydroxydopamine driven dopaminergic toxicity in an *in vivo* model of Parkinson’s disease (Darabi et al., 2018). These data suggests that SMER compounds enhance clearance via both degradation pathways, making VCP a particularly relevant target of interest. In terms of tractability, VCP is a well-known highly druggable AAA+ ATPase with well-defined nucleotide binding domains and an array of well-developed inhibitors (Chu et al., 2023). Wrobel et el demonstrate the potential of selectively modulating the D1 vs. the D2 domain which offers an attractive angle for medicinal chemistry optimization to separate the desired effects on proteostasis vs. the liabilities implicated for broad VCP peturbation (Wrobel et al., 2022). However, the potency and selectivity of SMER28 needs significant improvement as current concentrations used are in the micromolar range, raising the possibility of polypharmacology at clinically required doses (Tian et al., 2011; Wrobel et al., 2022). Safety issues may arise due to VCPs pleiotropic role in proteostasis and organelle quality control. ATPase activation may need to be titrated carefully to avoid chronic over activation which could result in altered turnover of essential proteins and an abnormally re-wired stress responses (Chu et al., 2023). Finally, patient stratification may be required where VCP mutations are present that change its ATPase activity, and so the tailored use of inhibitors or activators may be required accordingly (Wang et al., 2023; Ziff et al., 2023; Chu et al., 2023).

In 2025, Sedlacek et al. identified a class of novel NRF1 activators through targeted library screening. These NRF1 activators, in particular RUN-47, led to a stabilization of NRF1 and promoted an upregulation of UPS associated genes including proteasome 20S subunit β7, and increased the levels of autophagy receptor p62. The coordinated elevation of levels of proteasome and autophagy associated proteins translated to an increase in proteasome and autophagy activity, which reduced the number and size of neurodegenerative associated protein aggregates in cell models and in *C. elegans* (Sedlacek et al., 2025). This identification of selective Nrf1 activators support Nrf1 as a tractable upstream transcriptional regulator capable of simultaneously upregulating both protein clearance pathways. The translational challenges that remain with this target include how to sustain or carefully titrate Nrf1 activation for a therapeutic window without chronic proteasome hyperactivation *in vivo*, and how to address the context dependent effects of Nrf1 activation given Nrf1’s tight regulation by ER stress, proteasome function and cellular metabolic state. Further validation of these tool compounds in additional disease models and the development of biomarkers for Nrf1 target engagement will be critical to push NRF1 activators toward clinical trials (Łuczyńska et al., 2024).

NRF2 activators have demonstrated protective effects in several neurodegenerative models. Sulforaphane (SFN) is a natural product isolated from cruciferous vegetables that reacts with cysteine residues in Keap1, disrupting the Keap1-NRF2 interaction, leading to increased NRF2 stability and increased expression of UPS and autophagy related genes (Magesh et al., 2012). Lui and colleges demonstrated that SFN increased both proteasome and autophagy activity *in vitro* and *in vivo* and reduced mHtt accumulation and cytotoxicity in cell models of HD (Liu et al., 2014). Previous work has suggested that SFN increased β5 and Hsp27 expression and upregulated proteasome activity in cell cultures (Kwak et al., 2007; Gan et al., 2010). SFN improved cognition in AD mouse models (Kim et al., 2013), and shows protective capabilities in Parkinson’s disease models (Han et al., 2007; Jazwa et al., 2011), though outcomes vary with models and dosing. Other molecules such as resveratrol, quercetin, genistein and adrographolide have also been reported to activate NRF2 (Wong et al., 2018; Sedlacek et al., 2025), however, their pleiotropic activities and modest potency complicate analysis of Nrf2 specific driven effects. Dimethyl fumerate (DMF) is an FDA approved NRF2 activator for the treatment of multiple sclerosis that modulates redox responses and influences proteostasis regulators. DMF has been demonstrated to increase LC3 and ATG7 expression to promote autophagy activity in microglia, increase target protein degradation via the proteasome by downregulating DUBs and increase substrate ubiquitination and degradation (Dai et al., 2025; Lee et al., 2021). In preclinical PD models, DMF treatment protected dopaminergic neurons against alpha-synuclein toxicity in PD mouse models (Lastres-Becker et al., 2016), suggesting potential for repurposing DMF treatment for neurodegenerative settings. However, electrophilic NRF2 activators such as DMF can also engage off target cysteine residues and invoke Nrf2 independent effects (e.g., modulation of inflammatory signaling), which underscores the necessity for careful chemical design of Nrf2 activators and monitoring of Nrf2 target engagement biomarkers (Izumi and Koyama, 2024; Mayer et al., 2024). Omaveloxolone is a recent NRF2 small molecule activator approved for the treatment of Friedreich’s ataxia, but direct experimental evidence of its effect on UPS and autophagy flux is limited (Pilotto et al., 2024). Therefore, further work needs to be done to assess the meaningful effects of boosting protein clearance in neurodegeneration using this compound. Due to the combined significant effects on alleviating oxidative stress and promotion of protein clearance, the continued development of potent, selective and brain penetrant NRF2 activators represents an exciting opportunity to treat neurodegenerative diseases.

It is well established that mTOR inhibition leads to an induction of autophagy (Ali et al., 2022), and inhibition with small molecules, e.g., Torin1, can alleviate neuronal death in neurodegenerative disease models (Zhuang et al., 2020; Ali et al., 2022; Djajadikerta et al., 2020). mTOR inhibition has also been reported to increase the levels of protein degradation via the UPS, however, pinning down the effect of mTOR inhibition of UPS activity is complex and context dependent. Zhao et al demonstrated that mTOR inhibition via small molecule Torin1, increased overall cell proteolysis by both the UPS and autophagy. Notably, enhanced degradation of long-lived proteins via the UPS after Torin1 treatment was not due to increased proteasome catalytic activity, but a rapid upregulation of K48-linked ubiquitinated proteins, leading to increased substrate delivery to the proteasome (Zhao J. et al., 2015, Zhao and Goldberg, 2016). Conversely, Zhang et al., have reported that mTOR activation (whilst inhibiting autophagy) increases levels of active proteasomes through NRF1 induction and subsequent increase in proteasome subunit expression (Zhang et al., 2014). While these properties make mTOR an attractive dual regulator of autophagy and the UPS, its central role in growth, metabolism, and immune regulation presents significant translational challenges, including systemic toxicity and limited therapeutic windows with chronic inhibition (Davoody et al., 2024; Querfurth and Lee, 2021). Identification of more selective or context-restricted mTOR modulators may therefore be required to safely exploit this target.

Small molecule inhibitors of USP14, such as IU1 and its derivatives, enhance the degradation of pathological proteins via the UPS and autophagy. IU1 selectively binds to USP14 and blocks access of the C-terminus ubiquitin to the USP14 catalytic active site (Wang et al., 2018). Inhibition of the USP14 DUB trimming activities has been reported to consistently increase protein degradation via the proteasome across multiple systems by preventing premature deubiquitination of proteasome bound substrates, resulting in increased substrate commitment to degradation (Boselli et al., 2017; Hu et al., 2005; Kim et al., 2018; Lee B.-H. et al., 2010, Lim et al., 2024). In contrast, the effects of USP14 inhibition on autophagy appear to be more context dependent. While some studies report reduced autophagy flux under conditions of prolonged USP14 inhibition (Kim et al., 2018; Jha et al., 2025), the majority of pharmacological studies suggest that USP14 inhibition increases autophagy activity, potentially through altered ubiquitin signaling on autophagy regulators and enhanced turnover of inhibitory factors (Kim and Goldberg, 2018; Lim et al., 2024; Xu et al., 2016). These differences likely reflect variations in experimental context, including acute vs. chronic inhibition, proteasome-bound vs. free USP14 pools, and indirect effects driven by altered proteasomal flux. Despite this, USP14 inhibition has been demonstrated to promote the degradation of multiple neurodegenerative disease associated proteins, e.g., tau and TDP-43 (Lee B.-H. et al., 2010, Boselli et al., 2017) as well as increasing mitophagy in iNeurons (Bernardo et al., 2024), and PD mutant fruit fly models (Chakraborty et al., 2018), making it a potentially interesting therapeutic for AD, ALS/FTD and PD. However, some translational challenges remain including the need to improve the potency, selectivity and brain exposure of IU1 like compounds and define therapeutic windows to avoid chronic activation of degradation pathways.

Not all small molecules that modulate both pathways have a unified single target, meaning their effect may be driven by polypharmacology. For example, Fluspirilene, a small molecule calcium channel inhibitor, has been reported to activate both the UPS and autophagy to increase protein degradation (Xia et al., 2010; Fiolek et al., 2021). Xia et al reported that Fluspirilene binding to voltage-dependent calcium channel γ-1 subunit reduced calcium flux into cells and prevented the calpain1 mediated cleavage of Atg5, increasing the levels of full length Atg5 to bind Atg12/Atg16 for LC3-I to LC3-II processing (Xia et al., 2010). In a separate study, Fiolek and colleagues demonstrated that Fluspirilene and its analogs can directly bind to and activate the 20S proteasome overcoming inhibition induced by the accumulation of oligomeric alpha-synuclein (Fiolek et al., 2021). These types of molecules may serve as useful tools for assay development and screening, but due to high dosages required and off target effects they are of less interest for clinical pursuit.

## Therapeutic opportunities and challenges

While boosting the activities of the UPS and autophagy is an attractive therapeutic for decreasing pathogenic protein load in neurodegenerative diseases, target tractability varies markedly across pathway components. Core machinery of both systems are expressed ubiquitously, making it difficult to achieve neuronal specificity and raises concerns over global protein turnover imbalance. Although autophagy is often protective, persistent or excessive induction can trigger cell death, highlighting the importance of defining protective/destructive activation thresholds, treatment duration, and dosage when developing small molecule modulators (Orrenius et al., 2013). Similarly, chronic enhancement of UPS activity may impose an energetic burden or promote degradation of essential or long-lived proteins, potentially destabilizing the neuronal proteome. Moreover, most autophagy-enhancing compounds act indirectly through modulating mTOR and AMPK, leading to widespread metabolic effects beyond maintaining proteostasis (Alers et al., 2012). Neurons rely on precise spatial and temporal control of protein degradation, especially at synapses, where proteostasis is essential for cell signaling and plasticity, therefore overexpression or overactivation of the UPS in neurons may be detrimental to otherwise healthy neurons. Consistent with this, over expression of DUB USP7 in mice leads to abnormal neuronal migration and dendritic arborization, illustrating the sensitivity of neuronal architecture to imbalanced ubiquitin signaling (Qiao et al., 2022). However, neuronal specific PSMB5 subunit overexpression is neuroprotective in AD models of mice and Drosophila (Chocron et al., 2022), so the jury is still out on the safety of chronic overactivation of these systems. Indeed, given that overactivation of both pathways could exacerbate neuronal stress, one route to overcoming the safety of chronic hyperactivation of both systems could involve periodic dosing of these therapies to allow for a “proteasome/autophagy overactivation holiday” (Limanaqi et al., 2020; Barmaki et al., 2023).

As mentioned above, several pre-clinical studies provide strong proof-of-concept that enhancing the activities of the UPS and autophagy can reduce pathological protein aggregation and improve neuronal survival in animal models of neurodegenerative disease (Kim et al., 2013; Lastres-Becker et al., 2016; Darabi et al., 2018; Chakraborty et al., 2018). Despite this promising data, translation to the clinic has been limited. Reduction of pathological protein load does not always correlate with sustained functional or behavioral improvement, highlighting the limitations of current models and endpoints. Diagnosis of neurodegenerative diseases typically occurs after significant neuronal loss, reducing the likelihood that boosting proteostasis alone is enough to reverse neuronal damage. Therefore, early intervention is likely to play an important role in delaying disease progression. The development of validated robust biomarkers to monitor UPS and autophagy activity *in vivo* at early disease stages may be critical for patient stratification, target engagement assessment, and therapeutic timing. Longitudinal assessment of proteostasis capacity throughout disease progression and treatment will also be essential to balance efficacy against the inherent risks of globally enhancing cellular degradation pathways. Together, these considerations underscore that dual targeting strategies will likely require partial, temporally controlled modulation rather than sustained global activation of either pathway.

## Future work

Phenotypic screens are a key tool in drug discovery, allowing a large volume of target candidates to be assessed in a short time span (Carnero, 2006). Several screens have been run to try to elucidate new targets that will aid in decreasing protein inclusion formation and reducing toxic protein load in neurodegenerative models. It is unsurprising that in these screens, elements of both the UPS and autophagy consistently appear as hits, highlighting the importance of these two systems in disease, and their validity as targets (Kramer et al., 2018; Sweeney et al., 2024). However, few screens have focused on identifying modulators of both the proteasome and autophagy. In one example, Singh and colleges used a HEK293 cell line with a UPS reporter (Ub^G76V^-mCherry) and an autophagy reporter (GFP-LC3) in a CRISPR screen to identify genes for which knock down modulated both UPS and autophagy activity (Singh S. R. et al., 2021). From 80 hit candidates, Singh and colleges focused on Zinc finger protein 418 (Zfp418), knockdown of which altered the expression of several genes across the UPS and autophagy pathways including ubiquitinating and deubiquitinating enzymes and autophagy initiators (Singh S. R. et al., 2021).

The lack of overall data on mechanistic insights into regulating both pathways clearly demonstrates the requirement for further screening studies to identify novel pathways and drug targets. One of the key problems faced when using high-throughput screening in this area is the logistical need to operate low complexity screens with generic readouts of protein clearance as opposed to more in-depth mechanistic readouts at initial stages. Using simplistic protein clearance readouts in tissue culture rarely reflects the dynamic complexity of UPS and autophagy pathways in tissue and often leads to the detection of indirect hits. Further, the importance of the UPS and autophagy systems in cell health means many identified hits are associated with toxicity issues. The biology linking together the UPS and autophagy system in cellular models is incredibly complex, making mechanistic validation of hits difficult. As we have discussed in this review, multiple feedback mechanisms exist between these two systems, and this redundancy can make it difficult to identify unique regulators that are not part of a more generalized response. In addition, the temporal regulation of both the UPS and autophagy is incredibly dynamic, and temporal fluctuations make elucidating associated biological mechanisms of hits difficult. Going forward, advancement of better methods to read out crosstalk between the UPS and autophagy is need for a more granular understanding of novel mechanisms linking the two.

## Conclusion

Most neurodegenerative diseases are characterized by a build-up of pathological protein depositions, which lead to cell toxicity and widespread degeneration of neural tissue. There are currently very limited symptomatic or disease altering treatments, however, treatments that alter pathological protein aggregates have shown some success, e.g., monoclonal antibodies Lecanemab and Donanemab that reduce amyloid-beta burden and improves clinical outcomes in patients with Alzheimer’s disease (Van Dyck et al., 2023).

Therapeutics that aim to degrade pathological proteins, e.g., PROTACs, AUTACs, AUTOTACs etc., may not be as effective as initially hoped if the inherent ability of the cell to degrade misfolded proteins is impaired. Therefore, boosting the activities of the UPS and autophagy in neurodegenerative diseases may be a more viable therapeutic strategy, either alone or as a multi-modal treatment with protein degraders. In this review we have described several pathways that link the UPS and autophagy, which could be manipulated to boost the activity of both systems to increase protein degradation for pathological protein clearance in neurodegenerative disease. Some of these targets are more promising than others, with a key factor being the widespread and distinct activities of a single protein. For example, FOXO3 activation has the potential to increase the machinery expression and activity of both the UPS and autophagy, however, high levels of activation also increases the expression of proteins involved in apoptotic signaling. A second important factor that may limit target validation is the expression levels and function in disease, for example the VCP activator SMER28 appears promising in current research, however, loss of function mutations in VCP have been identified as causative in ALS/FTD and such therapies may be affected in the presence of mutations. The level and function of several UPS and autophagy machinery components have been reported to decline in aging and during diseases, therefore targets that boost activity without increasing expression of machinery proteins may need to be considered in combination with a treatment that boosts expression levels. Going forward, there is a clear need to identify novel targets with unaltered function or expression in aging and neurodegeneration and a well understood mechanism of action to reduce potential off-target effects. One promising strategy to identify these targets could be through conducting high throughput genetic phenotypic screening campaigns to identify a “super degrader” mechanistic pathway. The emergence of multiple modalities, from small molecules to protein degraders/molecular glues, could then be developed for this target/mechanistic pathway as the next novel therapeutic strategy to treat neurodegenerative proteinopathies.
